# Supplementary material for: Electrosynthesis of formamide from methanol and ammonia under ambient conditions
Source: Nat Commun. 2022 Sep 16;13:5452. doi: 10.1038/s41467-022-33232-w (PMC9481544; doi:10.1038/s41467-022-33232-w)
Supplement: Supplementary file 1 — Supplementary Information [file 41467_2022_33232_MOESM1_ESM.pdf]

Supplementary Information

Electrosynthesis of formamide from methanol and ammonia under ambient conditions

Meng et al.

Contents

Supplementary Figure 1. Production analysis.
Supplementary Figure 2. Formamide electrosynthesis performance from methanol and ammonia oxidation.
Supplementary Figure 3. Performance comparison.
Supplementary Figure 4. Performance comparison.
Supplementary Figure 5. Performance comparison.
Supplementary Figure 6. Characterization.
Supplementary Figure 7. Performance comparison.
Supplementary Figure 8. Performance comparison.
Supplementary Figure 9. Performance comparison.
Supplementary Figure 10. The stability measurement.
Supplementary Figure 11. The characterization of Pt-Ti after the stability measurement.
Supplementary Figure 12. Expandability to synthesize diverse amides.
Supplementary Figure 13. Setup scheme.
Supplementary Figure 14. The reaction network of methanol and ammonia under an applied potential of 1.68 V vs RHE on α-PtO_2.
Supplementary Figure 15. The reaction network of methanol and ammonia under an applied potential of 1.68 V vs RHE on β-NiOOH.
Supplementary Figure 16. The reaction network of methanol and ammonia under an applied potential of 1.68 V vs RHE on α-FeOOH.
Supplementary Figure 17. The reaction pathway and the energy diagram.
Supplementary Figure 18. The reaction pathway and the energy diagram.
Supplementary Figure 19. Different states.
Supplementary Figure 20. ^{1}H-NMR data.
Supplementary Figure 21. Setup scheme.
Supplementary Figure 22. In situ electrochemical FTIR spectra under different conditions.
Supplementary Figure 23. ^{1}H-NMR data.
Supplementary Figure 24. DEMS setup and data.
Supplementary Figure 25. DEMS and LC-MS data.
Supplementary Figure 26. LC-MS data.
Supplementary Figure 27. Techno-economic analysis.
Table S1. The binding energies of various adsorbates on α-PtO_2 surface with O stripe characterization.
Table S2. The binding energies of various adsorbates on the β-NiOOH(001) surface.
Table S3. The binding energies of various adsorbates on the α-FeOOH(001) surface.
Table S4. The charged states of the C atom and N atom in the relevant adsorbates on α-PtO_2, β-NiOOH, and α-FeOOH surfaces.

Table S5. The summarized properties of α-PtO_2, β-NiOOH, and α-FeOOH as the catalyst for the coupling of methanol and ammonia.

Table S6. Control experiments for exploring the reaction pathway.

Supplementary References (1-13)

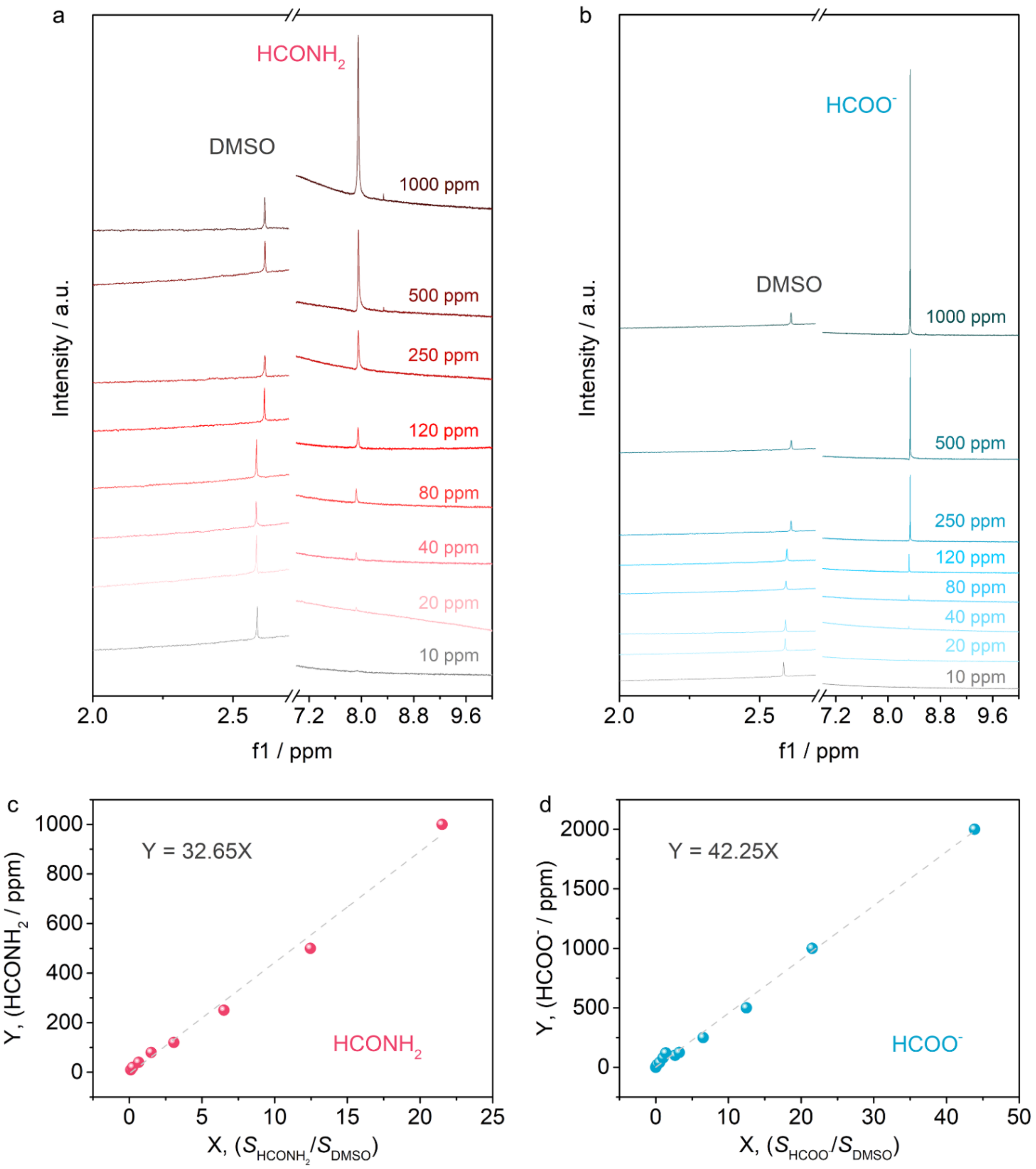

Supplementary Figure 1 │Production analysis. ^{1}H nuclear magnetic resonance (NMR) spectra of $HCONH_2$ (a) and $HCOO^-$ (b) with different concentrations. The standard curves of $HCONH_2$ concentration against X (X stands for the integral area ratio of $HCONH_2$/DMSO) (c) and $HCOO^-$ concentration against X (X stands for the integral area ratio of $HCOO^-$/DMSO) (d).

The standard curves of $HCONH_2$ and $HCOO^-$ both show the good linear relationship and follow the linear equation: $Y_{HCONH2} = 32.65\ X_{S(HCONH2)/S(DMSO)}$ and $Y_{HCOO-} = 42.25\ X_{S(HCOO-)/S(DMSO)}$, respectively.

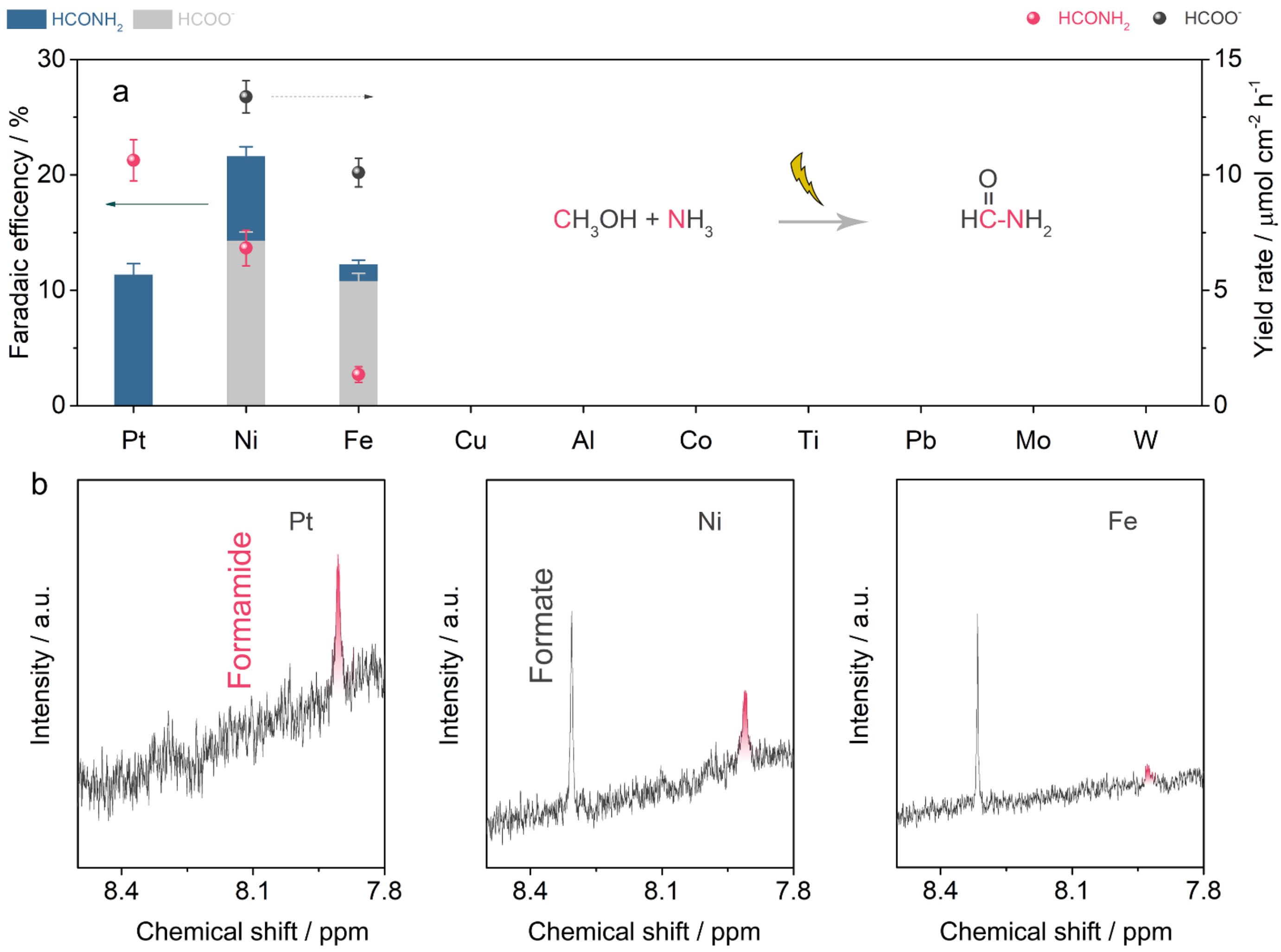

Supplementary Figure 2 │ Formamide electrosynthesis performance from methanol and ammonia oxidation. (a) The catalyst screening on formamide electrosynthesis at 10 mA cm^{-2}. (b) The typical ^{1}H-NMR spectra of the carbonaceous liquid products catalyzed by Pt, Ni, and Fe. Error bars correspond to the Standard Deviation (SD) of three independent measurements.

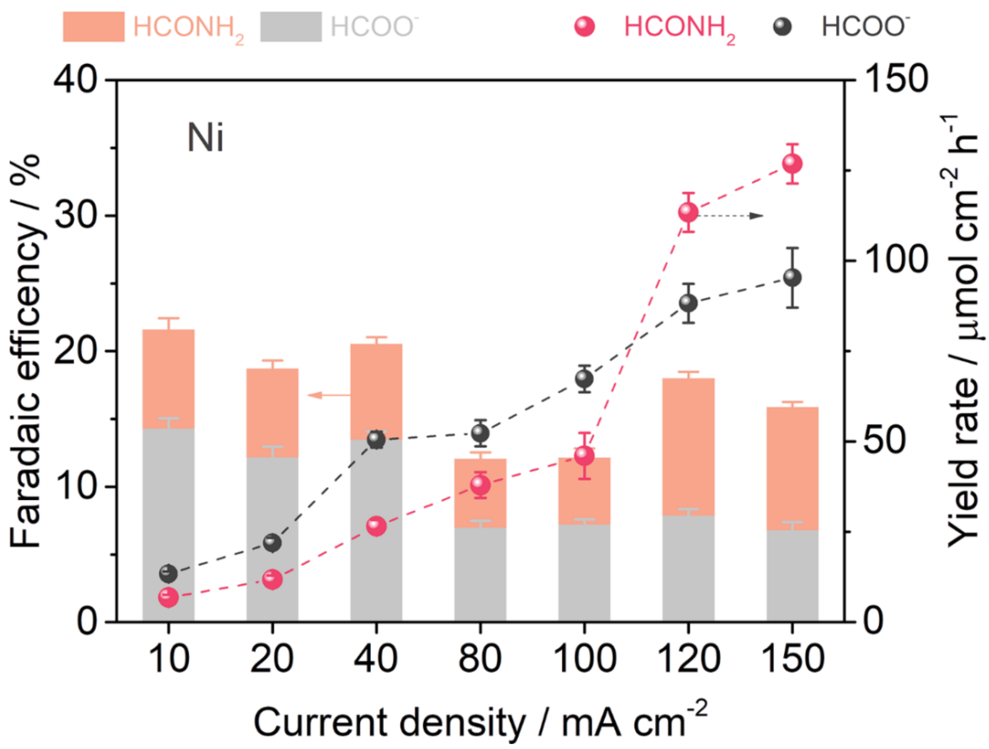

Supplementary Figure 3 │Performance comparison. Current density-dependent Faradaic efficiency and yield rate of $HCONH_2$ and $HCOO^-$ over Ni catalyst.

Ni possesses the capacity for $HCONH_2$ electrosynthesis while its Faradaic efficiency of $HCONH_2$ is ~7 % under different current densities. The $HCONH_2$ yield rate increases with the current density and reaches 126.8 μmol cm^{-2} h^{-1} at 150 mA cm^{-2}. In general, $HCOO^-$ product shows a similar tendency to that of $HCONH_2$. Error bars correspond to the SD of three independent measurements.

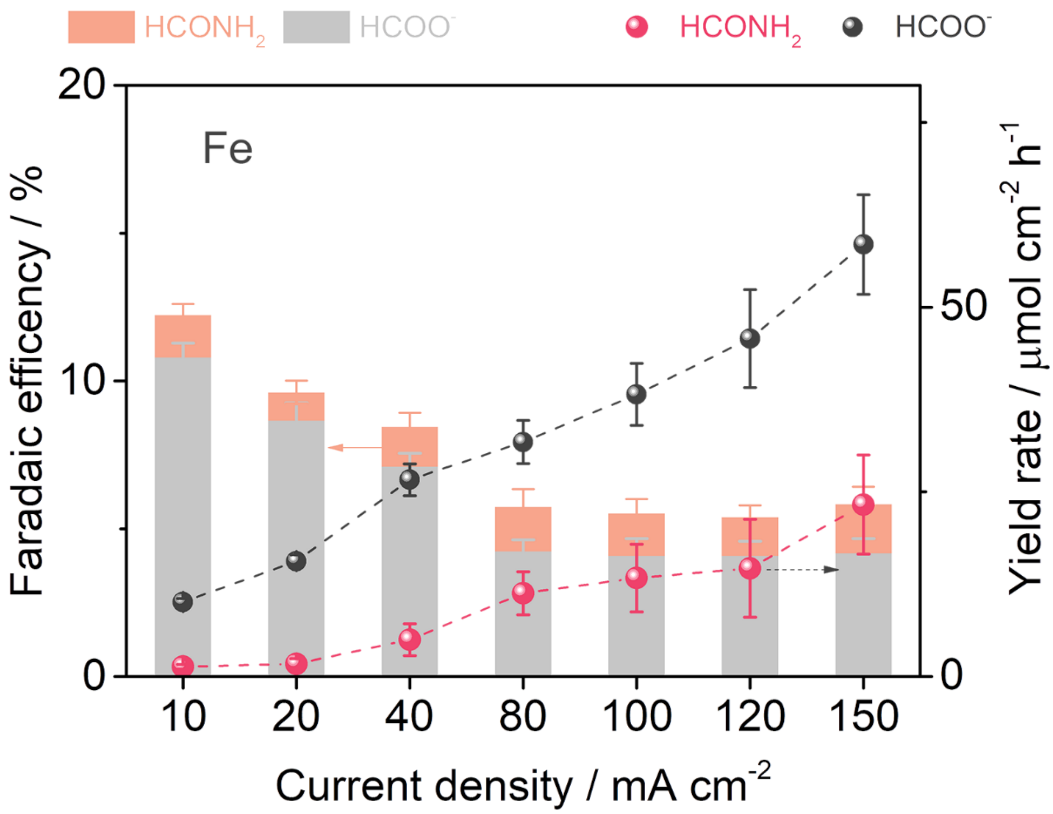

Supplementary Figure 4 │Performance comparison. Current density-dependent Faradaic efficiency and yield rate of $HCONH_2$ and $HCOO^-$ over Fe catalyst. Error bars correspond to the SD of three independent measurements.

Fe possesses the capacity for $HCONH_2$ electrosynthesis while its Faradaic efficiency of $HCONH_2$ is only ~2 % under different current densities. The $HCONH_2$ yield rate increases with the current density and reaches 23.28 μmol cm^{-2} h^{-1} at 150 mA cm^{-2}. In general, $HCOO^-$ product performance is better than $HCONH_2$.

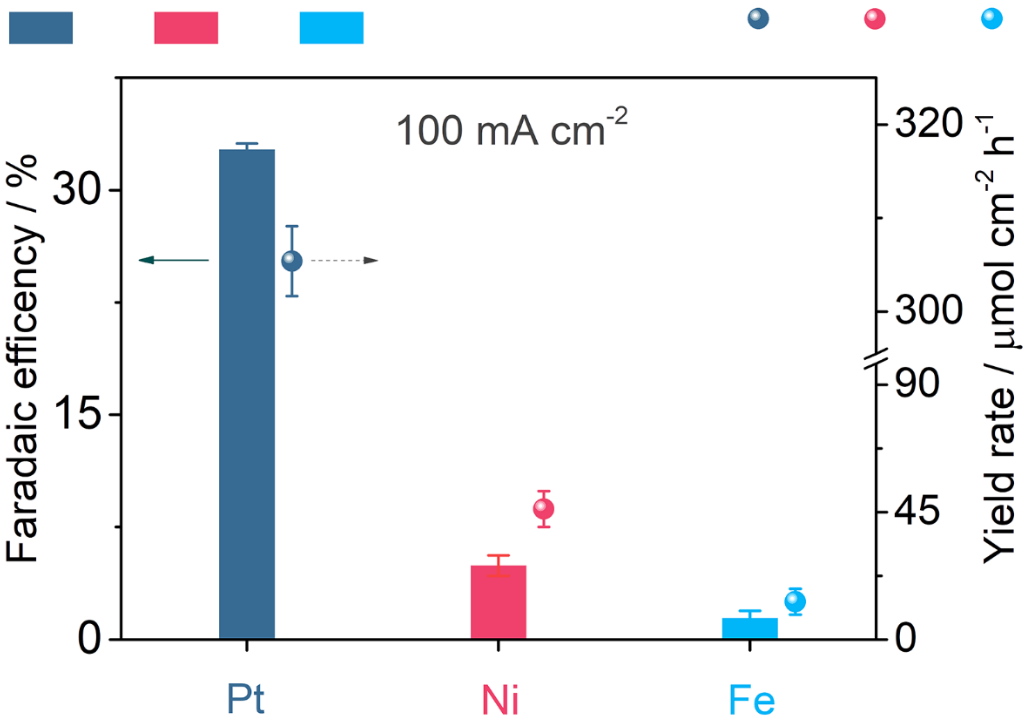

Supplementary Figure 5 │Performance comparison. The performance comparison among Pt, Ni, and Fe for $HCONH_2$ electrosynthesis at the current density of 100 mA cm^{-2}. Error bars correspond to the SD of three independent measurements.

Compared with Ni and Fe, Pt shows higher activity for $HCONH_2$ electrosynthesis. Its Faradaic efficiency (32.70 %) is nearly 6.6 and 22.9 times as high as those of Ni (4.93 %) and Fe (1.43 %).

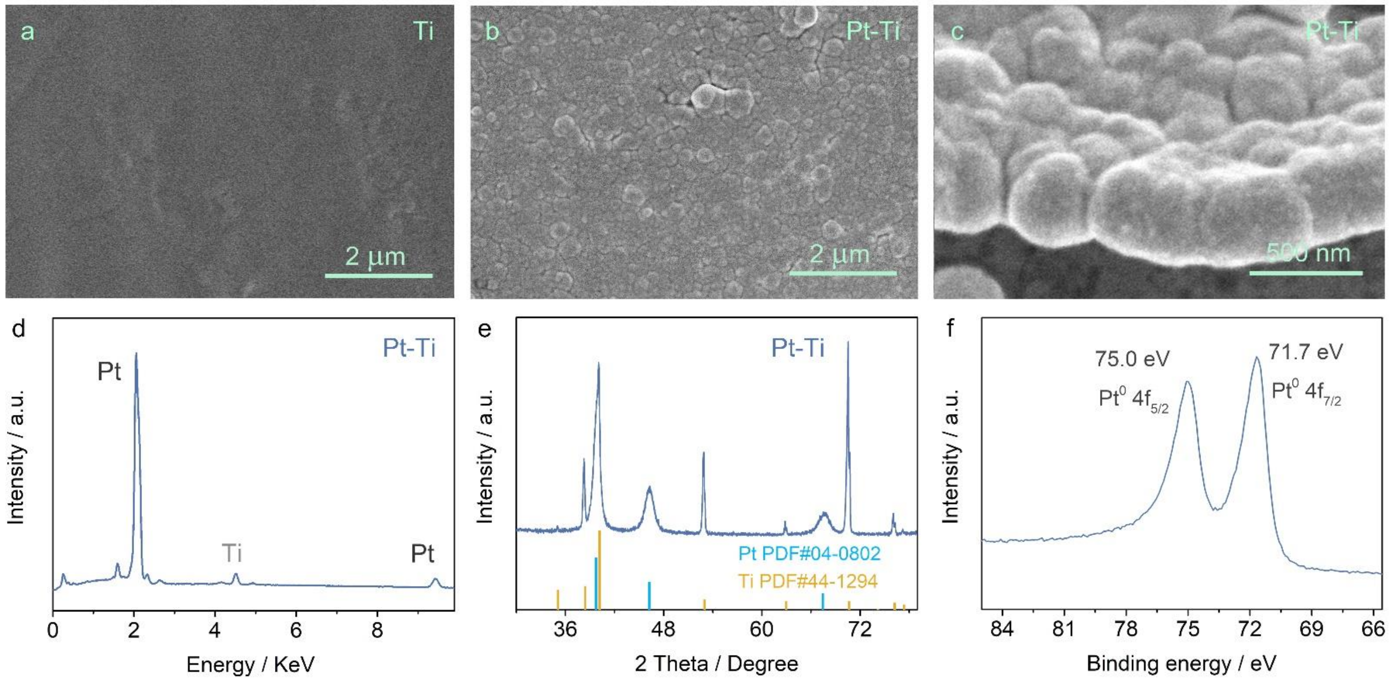

Supplementary Figure 6 │Characterization. SEM images of Ti (a) and Pt-Ti (b, c). EDX spectrum (d) and XRD pattern (e) of Pt-Ti. XPS spectrum of Pt in Pt-Ti (f).

Ti substrate shows a smooth surface (Supplementary Fig. 6a) and becomes rough after the electrodeposition of Pt (Supplementary Fig. 6b). A cross-section SEM image indicates a compact Pt film with a thickness of ~ 0.48 μm on Ti substrate (Supplementary Fig. 6c). EDX spectrum depicts the elements of Pt and Ti in the Pt-Ti sample (Supplementary Fig. 6d). XRD pattern of Pt-Ti shows the sample is composed of Pt (PDF # 04-0802) and Ti (PDF # 44-1294) (Supplementary Fig. 6e). XPS spectrum proves the existence formation of metallic Pt^0 in Pt-Ti (Supplementary Fig. 6f).

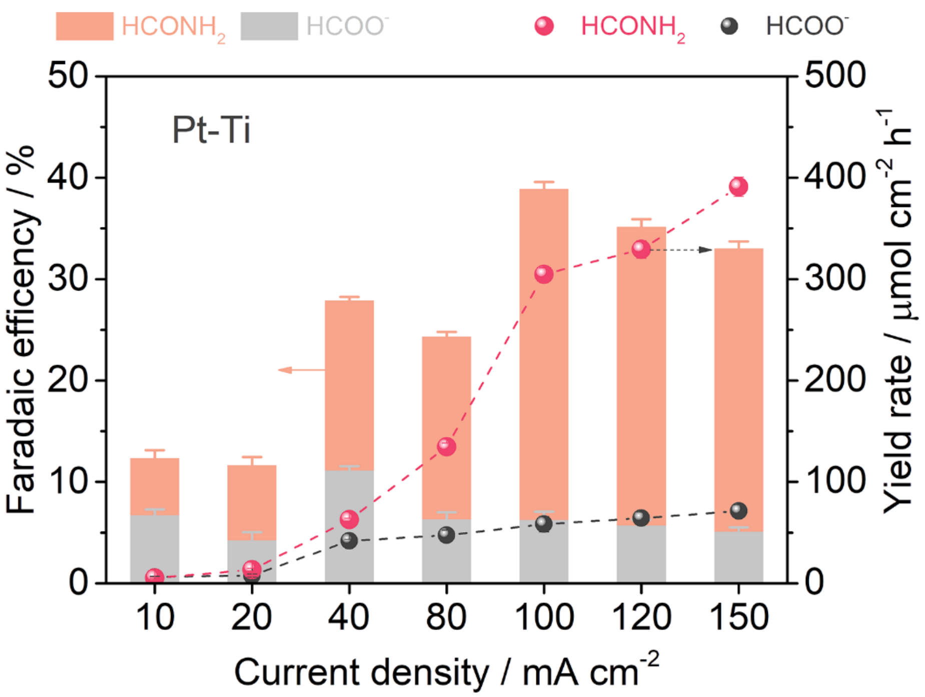

Supplementary Figure 7 │Performance comparison. Current density-dependent Faradaic efficiency and yield rate of $HCONH_2$ and $HCOO^-$ over Pt-Ti catalyst. Error bars correspond to the SD of three independent measurements.

The Faradaic efficiency of $HCONH_2$ shows volcanic curves with the current density and reaches the maximum value of 32.64 % at a current density of 100 mA cm^{-2}. The $HCONH_2$ yield rate keeps increasing with the current density and reaches the maximum value of 390.99 μmol cm^{-2} h^{-1} at a current density of 150 mA cm^{-2}. In general, $HCOO^-$ product performance maintains at a low level.

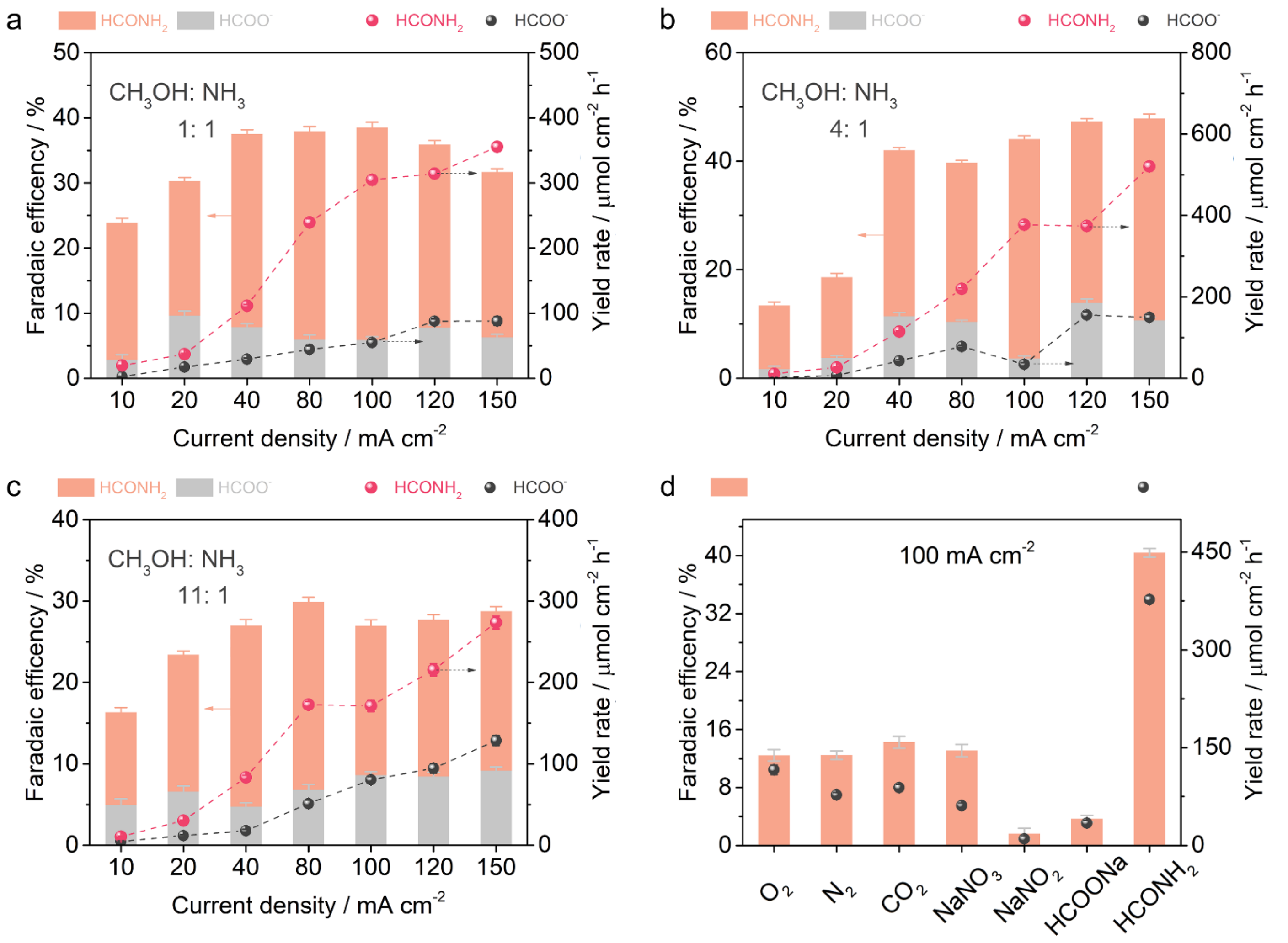

Supplementary Figure 8 │Performance comparison. Current density-dependent Faradaic efficiency and yield rate of $HCONH_2$ and $HCOO^-$ over Pt-Ti catalyst with different volume ratios of CH_3OH to NH_3: 1:1 (a), 4:1 (b), and 11:1 (c). The Faradaic efficiencies and yield rates of various products over Pt-Ti in a 4:1 volume ratio of CH_3OH to NH_3 at 100 mA cm^{-2} (d). Error bars correspond to the SD of three independent measurements.

The optimal volume ratio of CH_3OH to NH_3 is 4:1.

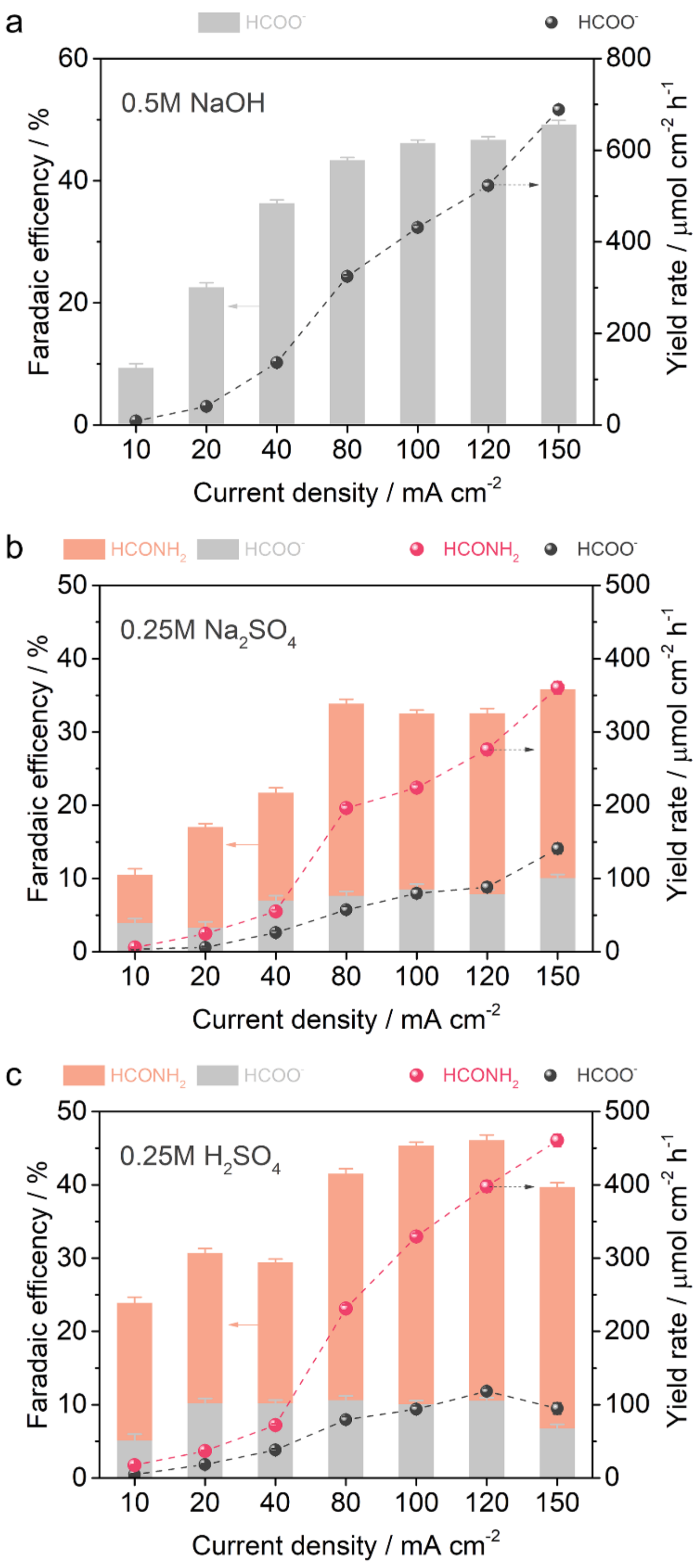

Supplementary Figure 9 | Performance comparison. Current density-dependent Faradaic efficiency and yield rate of $HCONH_2$ and $HCOO^-$ over Pt-Ti catalyst under different pH conditions: 0.5M NaOH (a), 0.25M Na_2SO_4 (b), and 0.25M H_2SO_4 (c). Error bars correspond to the SD of three independent measurements.

As shown in Figure 2d and Supplementary Fig. 9, in 0.5M NaOH aqueous solution, the carbonaceous liquid product was only formic acid and the formamide product was totally suppressed. It was probably aroused by the decomposition of formaldehyde, the intermediate of CH_3OH oxidation in a strong alkali solution via the Cannizzaro reaction. The acid (0.25M H_2SO_4) and natural (0.25M Na_2SO_4) solutions were beneficial for formamide generation.

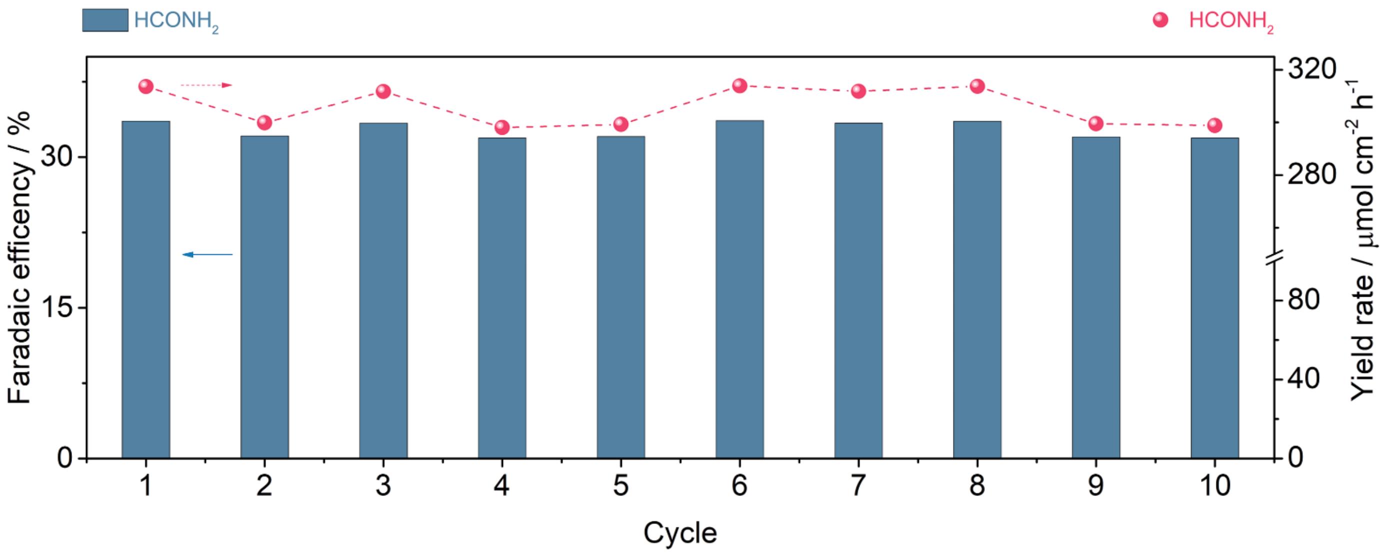

Supplementary Figure 10 │The stability measurement. The stability measurement of Pt-Ti for formamide electrosynthesis using a 2:1 volume ratio of CH_3OH to NH_3 in 0.5M $NaHCO_3$ at 100 mA cm^{-2}.

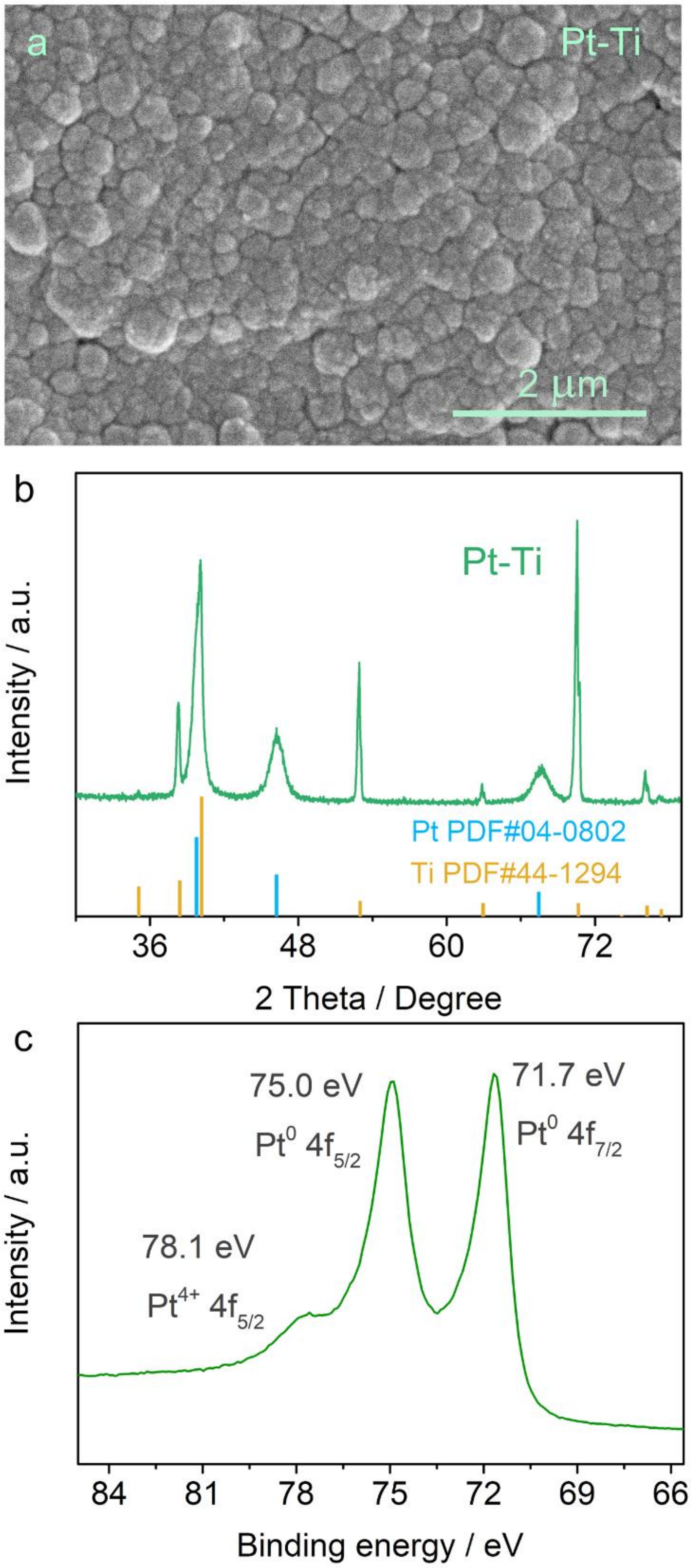

Supplementary Figure 11 │The characterization of Pt-Ti after the stability measurement. SEM image (a), XRD pattern (b), and XPS spectrum (c).

After the stability measurement, the morphology of the Pt-Ti catalyst maintains well. The crystalline structure of Pt-Ti is still composed of Pt (PDF # 04-0802) and Ti (PDF # 44-1294), while Pt^{4+} appears on the surface.

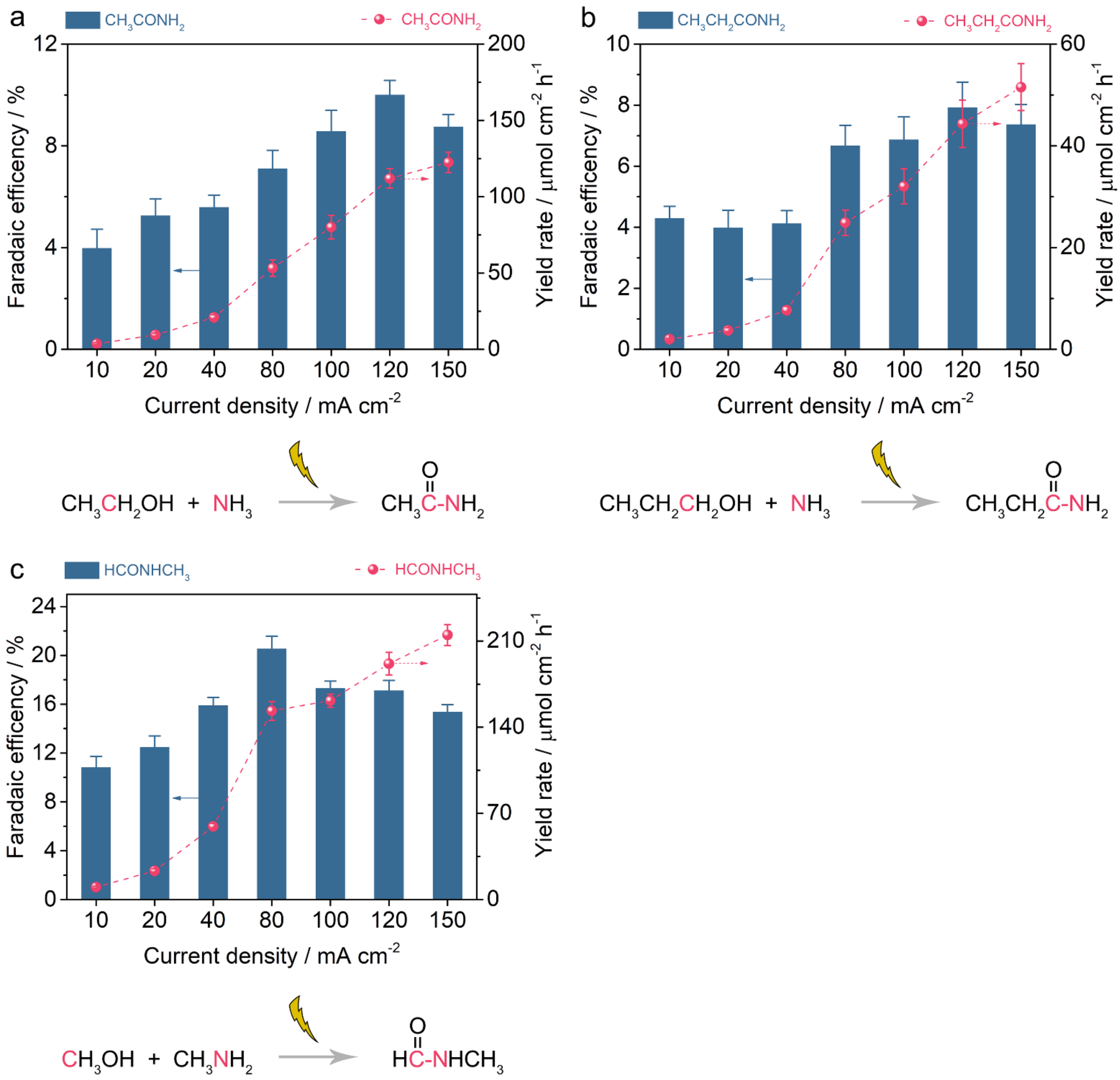

Supplementary Figure 12 │Expandability to synthesize diverse amides. Current density-dependent Faradaic efficiency and yield rate of (a) CH_3CONH_2, (b) $CH_3CH_2CONH_2$, and (c) $HCONHCH_3$ over Pt-Ti catalyst. Error bars correspond to the SD of three independent measurements.

The one-pot electrolysis approach can be expanded to synthesize other amides such as CH_3CONH_2, $CH_3CH_2CONH_2$, and $HCONHCH_3$. After optimizing the current density, the highest Faradaic efficiencies can reach 10.01 % for CH_3CONH_2, 7.93 % for $CH_3CH_2CONH_2$, and 20.54 % for $HCONHCH_3$.

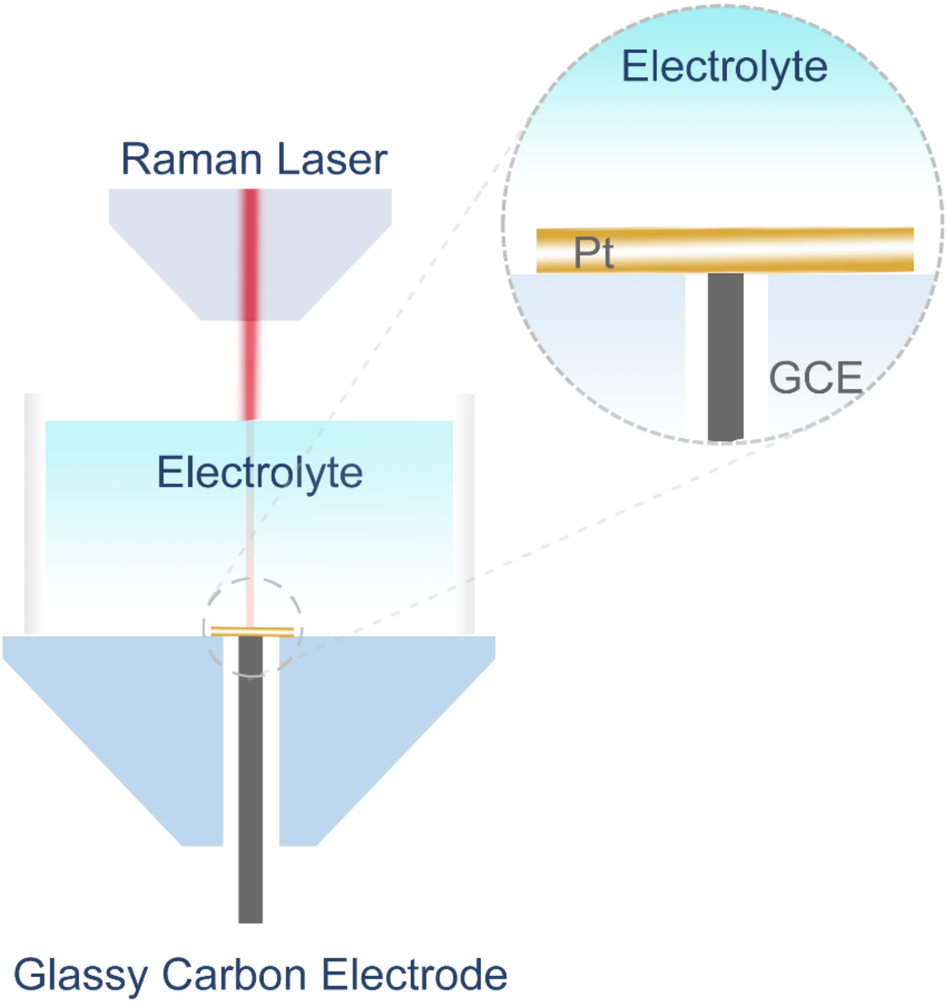

Supplementary Figure S13 │Setup scheme. Schematic illustration for in situ Raman electrochemical measurement.

Theoretical Methods

All DFT calculations were performed with the VASP code with the Perdew-Burke-Ernzerhof (PBE) exchange-correlation functional[4,5]. Projector Augmented Wave (PAW) potentials were used to describe ionic cores[6]. The adsorbates were free to move in all directions. The atomic relaxations were carried out with the quasi-Newton minimization scheme until the maximum force on any atom was below 0.05 eV $Å^{-1}$. The vertical separation between periodically repeated images was in all cases more than 15 Å and dipole corrections were applied. The geometry optimizations were performed with a plane-wave cutoff of 450 eV, 400 eV, and 450 eV for α-PtO_2, β-NiOOH, and α-FeOOH, respectively. The Brillouin zones of all systems were sampled with Monkhorst-Pack grids[7]. The k-point sampling (k_1, k_2, k_3) was such that the product between its components and the norms of the supercell vectors (a, b, c) was at least (25 Å, 25 Å, 25 Å), which ensures that the meshes are dense enough for adsorption energies to be converged within a range of 0.05 eV or below. The Fermi level was smeared with the Methfessel-Paxton approach with a Gaussian width of 0.2 eV, and all energies were extrapolated to T = 0 K[8]. To correct the overdelocalization errors of electrons in FeOOH and NiOOH, DFT+U approach was employed and U-J values of 5.0 eV and 5.5 eV was added to PBE functionals for FeOOH and NiOOH[9], respectively. The binding energies were calculated relative to the corresponding clean surfaces and gas-phase references. The following equation explains the calculation process with $*NH_x$ as an example.

$$\Delta E_{\mathrm{ad,*NH}_x} = E_{*\mathrm{NH}_3} - E_{*} - (E_{\mathrm{NH}_3} - ((3-x)/2)E_{\mathrm{H}_2})$$

The gas-phase references were calculated in cubic boxes of 15 Å × 15 Å × 15 Å using a gamma point distribution and an electronic temperature of 0.001 eV. To convert DFT-calculated energies into free energies, we added entropy and zero-point energy corrections to the gas-phase references, taking into account that $G = E_{\mathrm{DFT}} + ZPE - T*S$. The zero-point energies of gases and the vibrational entropies of adsorbates were calculated through vibrational-frequency analysis using the harmonic oscillator approximation. Taking into account the difference between the standard formation energies of the gas phase and liquid phase water, $G_{H2O(l)}$ is corrected by -0.09 eV from $G_{H2O(g)}$. The solvation effect of water is included in all the simulations of surfaces and adsorbates through the implicit solvation model and VASPSol code developed by Hennig *et al.*[10,11] and the solvation energy is taken into account in Gibbs free energy of adsorbates and reactions.

As indicated by Raman's characterization and previous works, the crystal structure of α-PtO_2 with the top oxygen atoms in the stripe characterization was built. Combination of the thermodynamically most stable surface with Raman characterization, (001) and (100) crystallographic planes were used as the model surfaces for β-NiOOH and α-FeOOH, respectively.

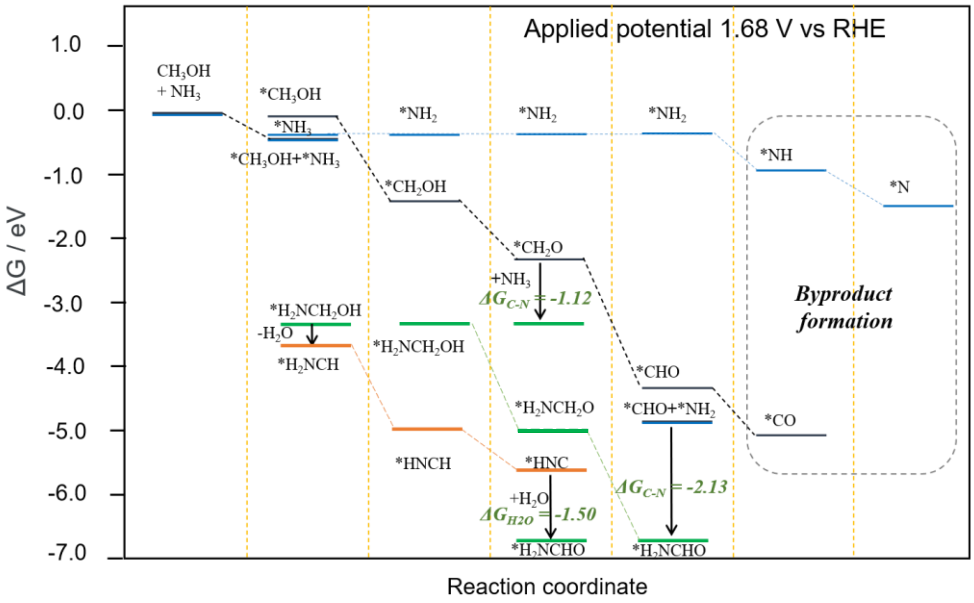

Supplementary Figure 14 │The reaction network of methanol and ammonia under an applied potential of 1.68 V vs RHE on α-PtO_2. The formation of *NH and *CO leads to the byproduct generation of N_2, nitrite, nitrate, CO_2, and acetate. All the reaction steps connected by dotted lines are the electrochemical dehydration processes with the transfer of (H^+ + e).

Since methanol and ammonia coupling reaction takes place under highly oxidized potentials (> ~1.5 V vs. RHE) and the hydrogenation through the transfer of (H^++e) would be hardly possible, we consider once the formation of *NH and *CO, there is no chance for such species to participate in the C-N bond making steps but only byproduct formation.

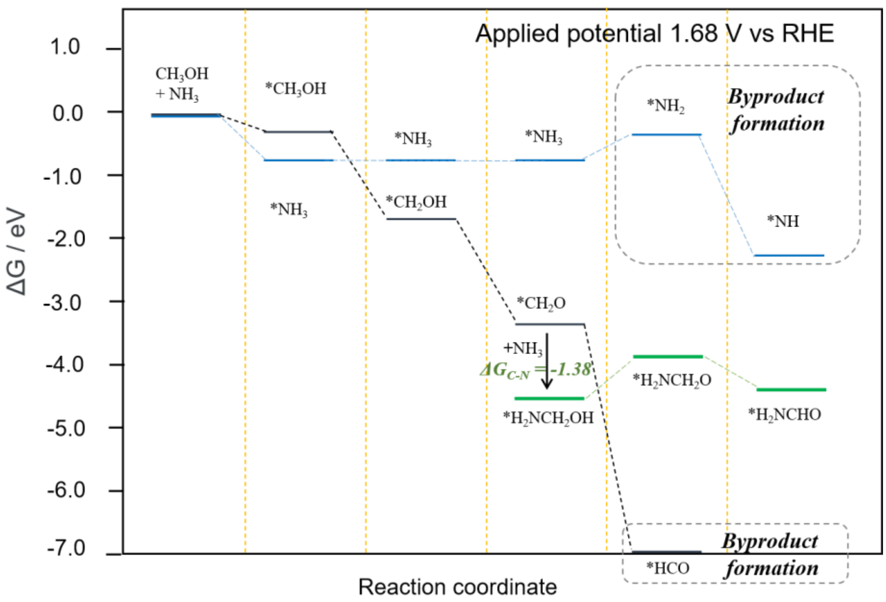

Supplementary Figure 15 │The reaction network of methanol and ammonia under an applied potential of 1.68 V vs RHE on *β*-NiOOH. The formation of *NH and *HCO leads to the byproduct generation of N_2, nitrite, nitrate, CO_2, and acetate. All the reaction steps connected by dotted lines are the electrochemical dehydration processes with the transfer of (H^+ + e).

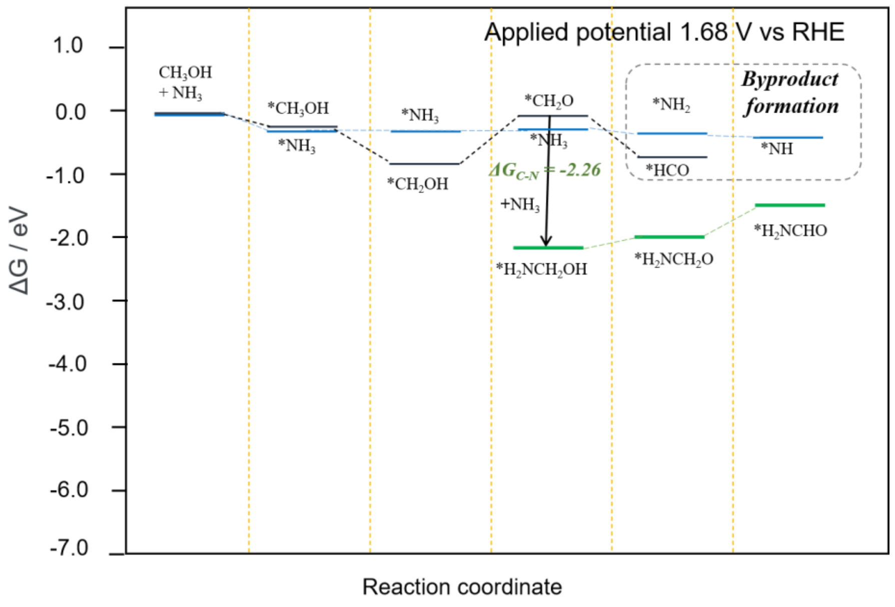

Supplementary Figure 16 │The reaction network of methanol and ammonia under an applied potential of 1.68 V vs RHE on α-FeOOH. The formation of *NH and *HCO leads to the byproduct generation of N_2, nitrite, nitrate, CO_2, and acetate. All the reaction steps connected by dotted lines are the electrochemical dehydration processes with the transfer of (H^+ + e).

The reaction network including various electrochemical dehydrogenation steps as well as C-N bond formation steps are presented under a uniform applied potential of 1.68 V vs RHE, at which the generation of *NH_2 on α-PtO_2 becomes thermodynamically feasible. It could be noticed through Supplementary Figs. 14-16 that the production of formamide has the highest thermodynamic driving force on α-PtO_2 and the lowest driving force for byproduct formation, which are summarized in Supplementary Table S5.

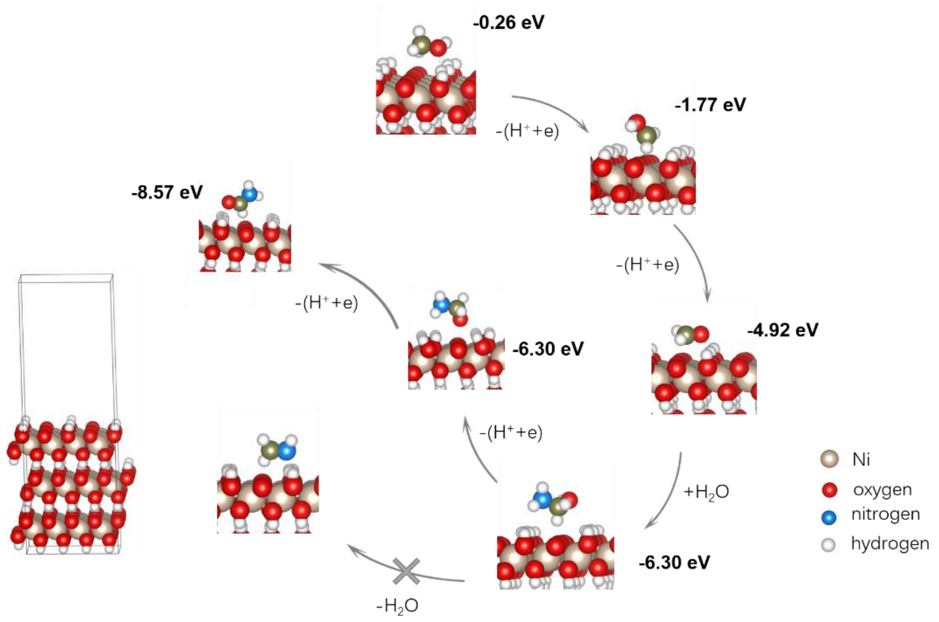

Supplementary Figure 17 │The reaction pathway and the energy diagram. The reaction pathway and the energy diagram for formamide formation on β-NiOOH at applied potentials of 2.51 V vs RHE. All steps in the shown pathway are thermodynamically feasible.

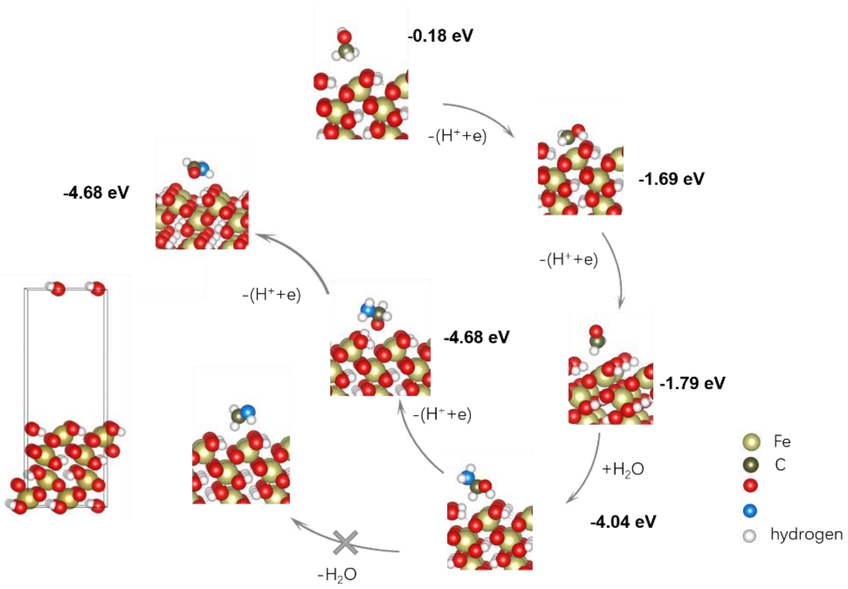

Supplementary Figure 18 │The reaction pathway and the energy diagram. The reaction pathway and the energy diagram for formamide formation on α-FeOOH at applied potentials of 2.57 V vs RHE. All steps in the shown pathway are thermodynamically feasible.

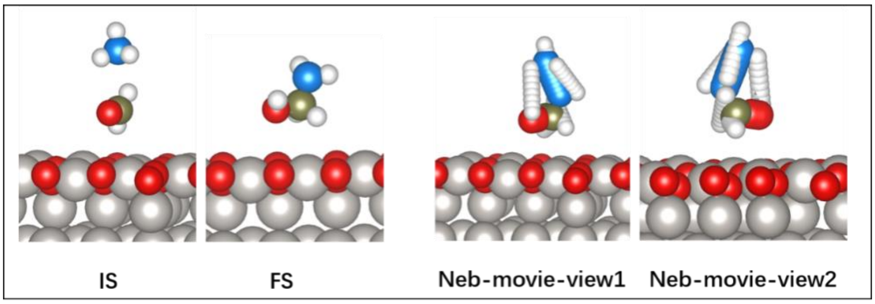

Supplementary Figure 19. Different states. The initial state (IS), final state (FS), and movie images for searching transition states using the NEB method.

For the possible C-N bond-making steps (*CH_2O+NH_3=*CH_2OHNH_2), we have tried to implement the typical Nudged Elastic Band (NEB) and/or ci-NEB method for searching transition states and calculating barrier energies. Supplementary Fig. 19 showed the movie-images of the barrier path with *CH_2O+NH_3 on α-PtO_2 surface as an example. However, these calculations hardly converge. The weak interaction between *CH_2O/NH_3 and the surfaces means that the image-species, especially the H atom that transfers from NH_3 to CH_2O have very weak sensitivity to the surface electronic structures as well as have multi-dimensional freedom to move, which twists the whole strings during the calculations. The other reason for the failure in convergence could be the difference between IS and FS are relatively large (minimum 6 images to be inserted to give a reasonable path). Therefore, we give up the NEB methods to calculate barrier energies. Instead, the C-N bond-making step between *CH_2O and NH_3 is the nucleophilic attack process, i.e., the positively charged C is attacked by the electronegative N atom in NH_3[1]. Therefore, the charged states of C in *CH_xO and N in NH_3/NH_2 qualitatively demonstrate the feasibility of the C-N bond-making process. The charge analysis of the relevant adsorbates is done using Bader charge analysis. The charged states of the C atom and N atom are presented in Table S4.

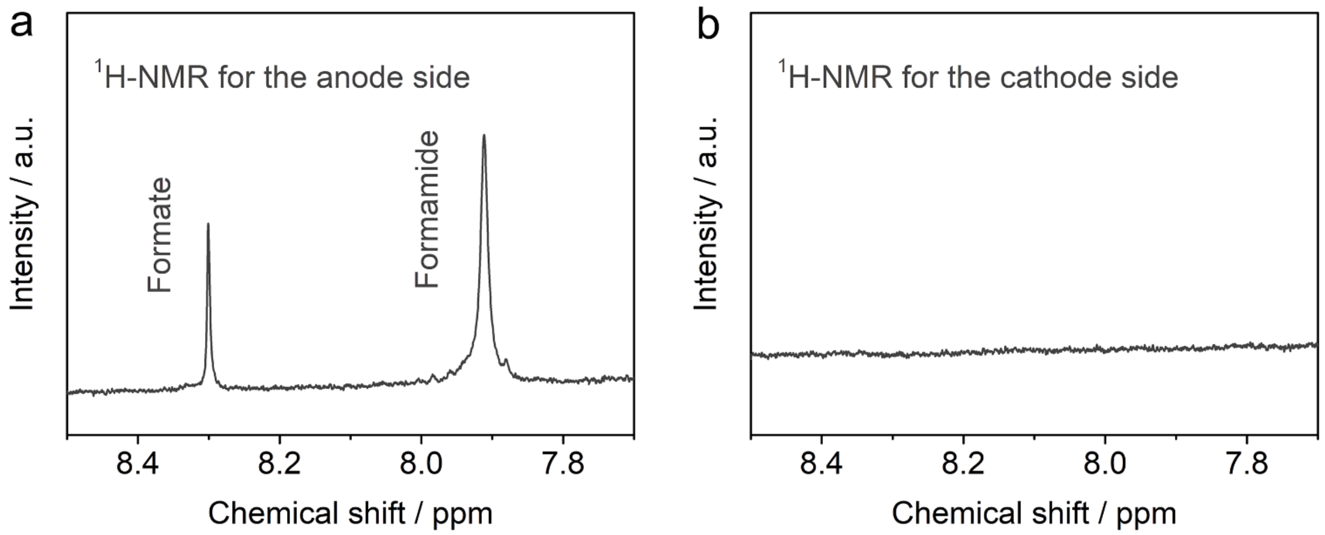

Supplementary Figure 20 │^{1}H-NMR data. ^{1}H-NMR spectra for formamide electrosynthesis in the different chambers of an H-type cell separated by a Nafion membrane after the electrolysis at 100 mA cm^{-2} current density.

A two-chamber (H-Cell) separated by a Nafion membrane is used for studying the effect of the cathode reactivity for formamide synthesis and both sides are a mixture of CH_3OH and NH_3 in 0.5M $NaHCO_3$. Both cathode and anode are Pt-Ti foil. After a three-hour electrolysis at 100 mA cm^{-2} current density, formamide can only be detected at the anode side (Supplementary Fig. 20). The result indicates the cathode reactivity does not affect formamide formation and formamide is generated via the electrooxidation reaction**.**

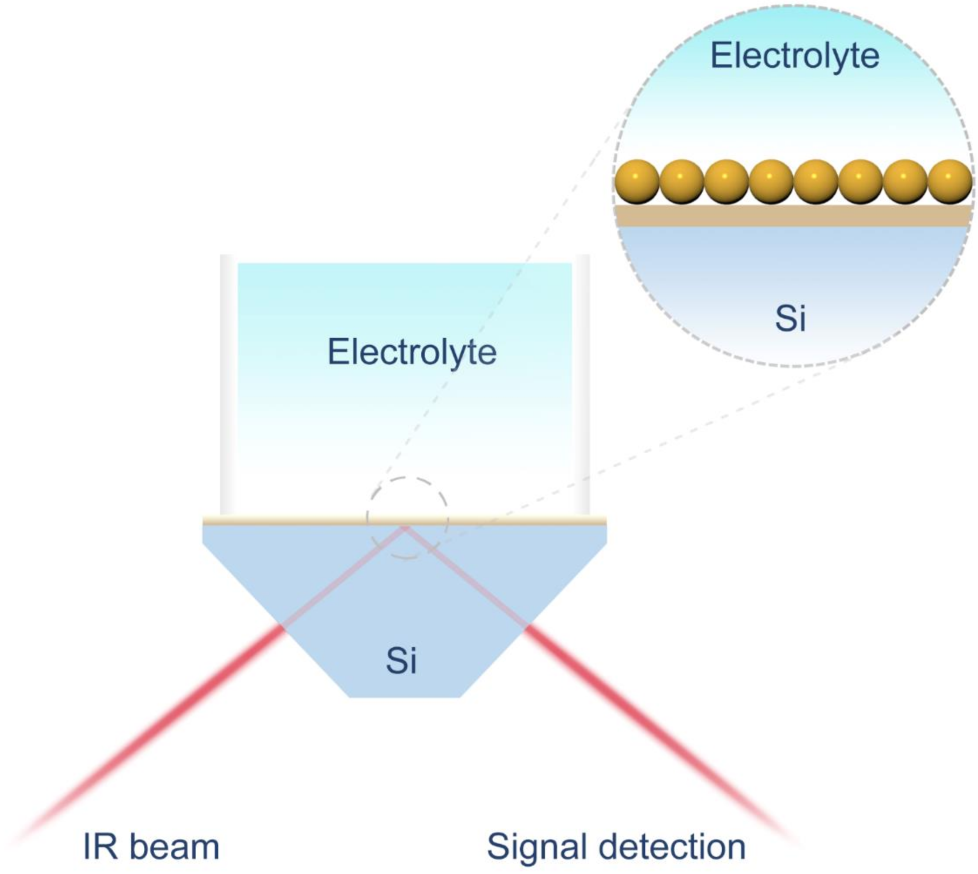

Supplementary Figure S21 │Setup scheme. Schematic illustration for in situ ATR-FTIR electrochemical measurement.

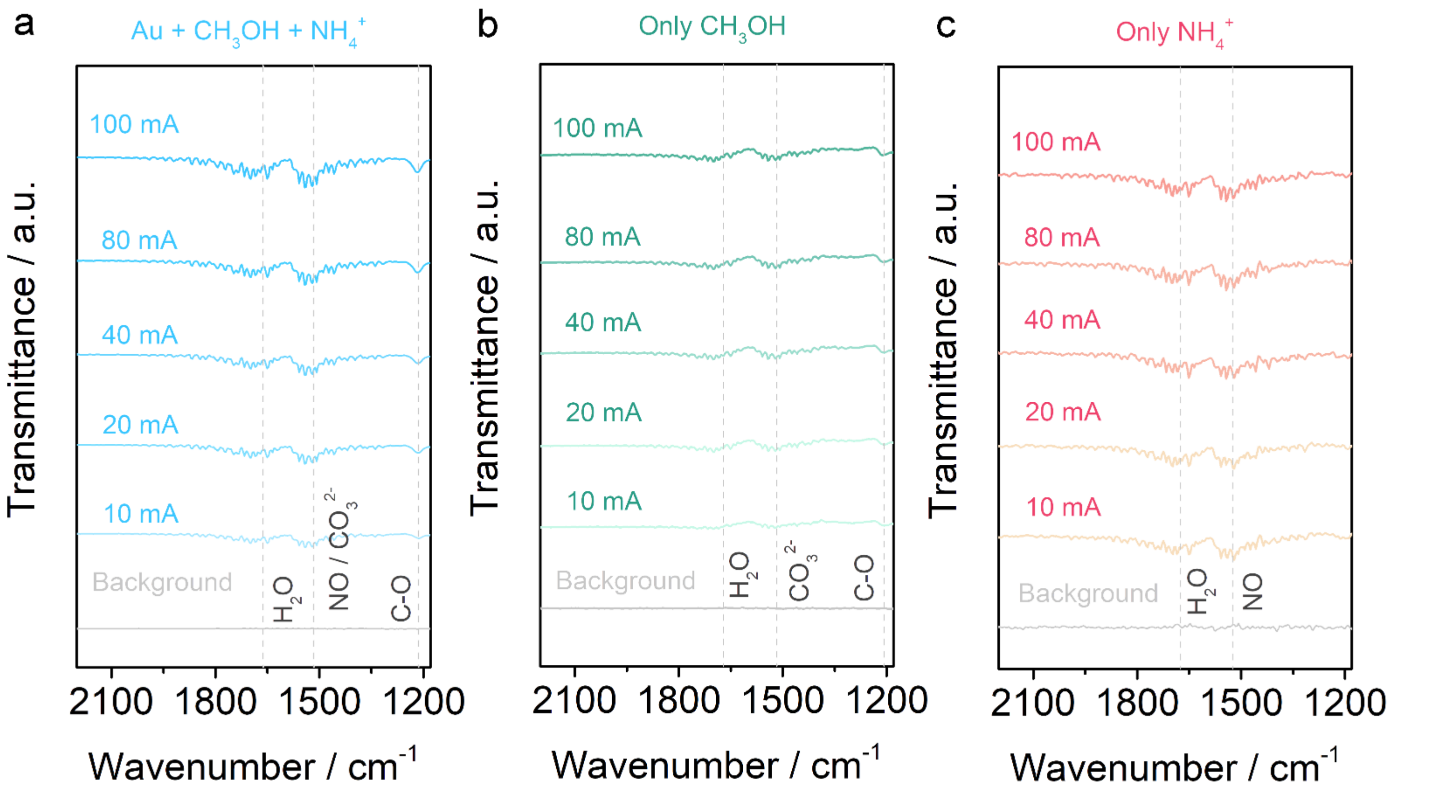

Supplementary Figure 22 │In situ electrochemical FTIR spectra under different conditions. Au substrate for methanol and ammonia electrooxidation (a), CH_3OH electrooxidation (b) and NH_4^+ electrooxidation on Pt (c).

To illustrate the influence of Au substrate on the FTIR peak, Au-coated Ti foil is synthesized using the same method as Pt-coated Ti foil to test its performance for formamide electrosynthesis at 100 mA cm^{-2} current density. As shown in Supplementary Fig. 23, no formamide is found after a three-hour reaction, suggesting its inert activity for formamide synthesis. Furthermore, Au-coated Si substrate is directly carried out for in situ FTIR (Supplementary Fig. 22a), water peak (~1680 cm^{-1}), NO/CO_3^{2-} (~1520 cm^{-1}) and C-O (1220 cm^{-1}) bond are well recognized and no C-N is found[2,3]. Hence, the influence of Au substrate on the C-N signal can be excluded.

For in situ FTIR measurements of single CH_3OH (Supplementary Fig. 22b) or NH_3 (Supplementary Fig. 22c) electrooxidation, CO_3^{2-} and C-O bonds are detected during methanol electrooxidation, and NO is detected during NH_3 electrooxidation.

Those results prove the FTIR peaks of Au substrate and single CH_3OH or NH_3 electrooxidation do not disturb the detection of C-N and C≡N.

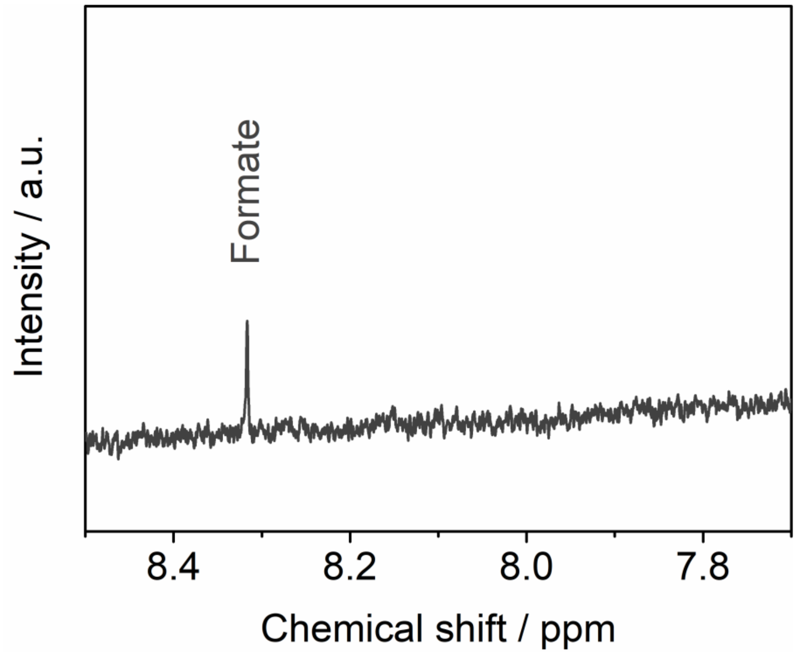

Supplementary Figure 23 │^{1}H-NMR data. ^{1}H-NMR spectrum for formamide electrosynthesis using Au-Ti.

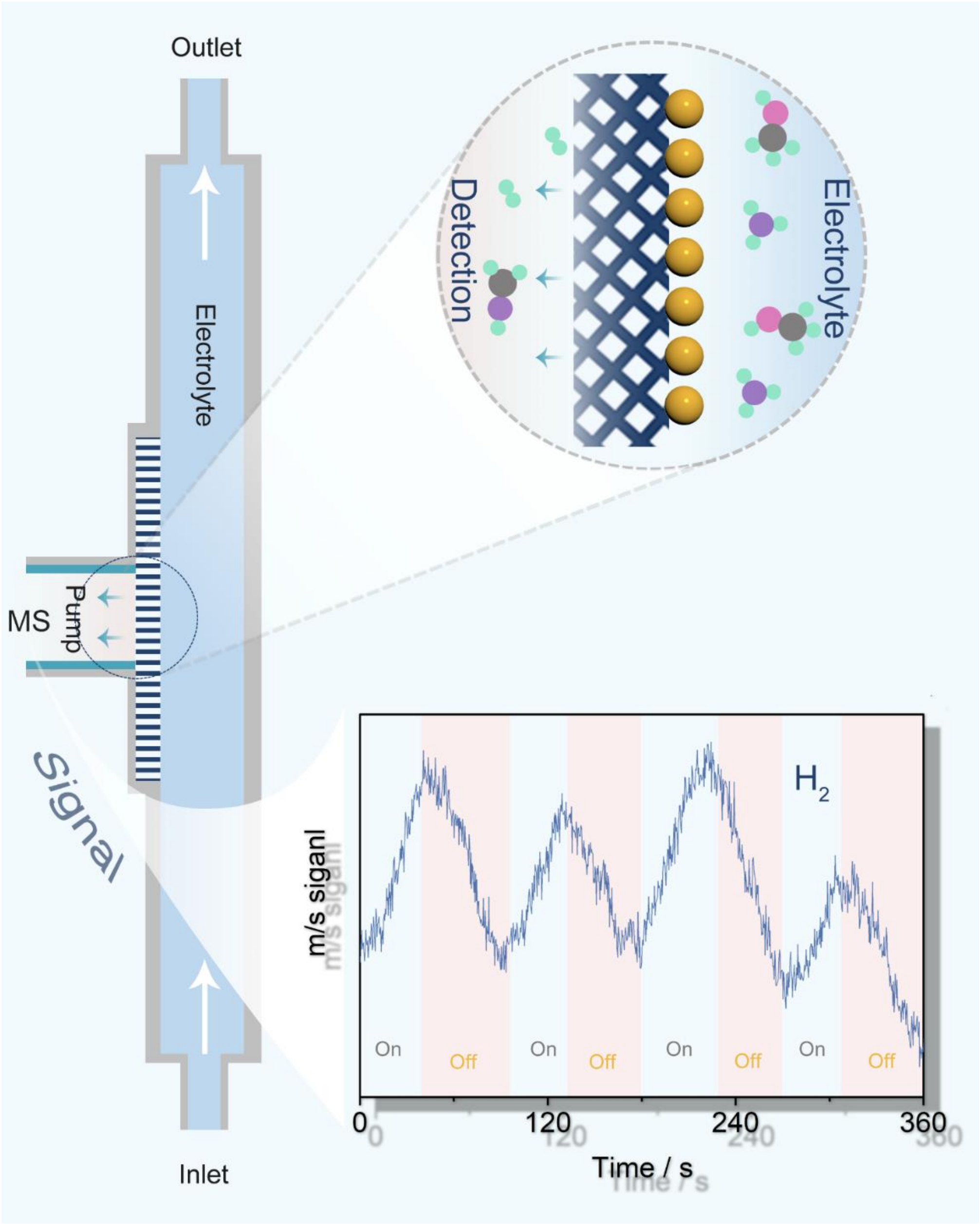

Supplementary Figure S24 │DEMS setup and data. Schematic illustration for the online DEMS electrochemical measurement and the corresponding DEMS spectrum of H_2 generation in the cathode zone.

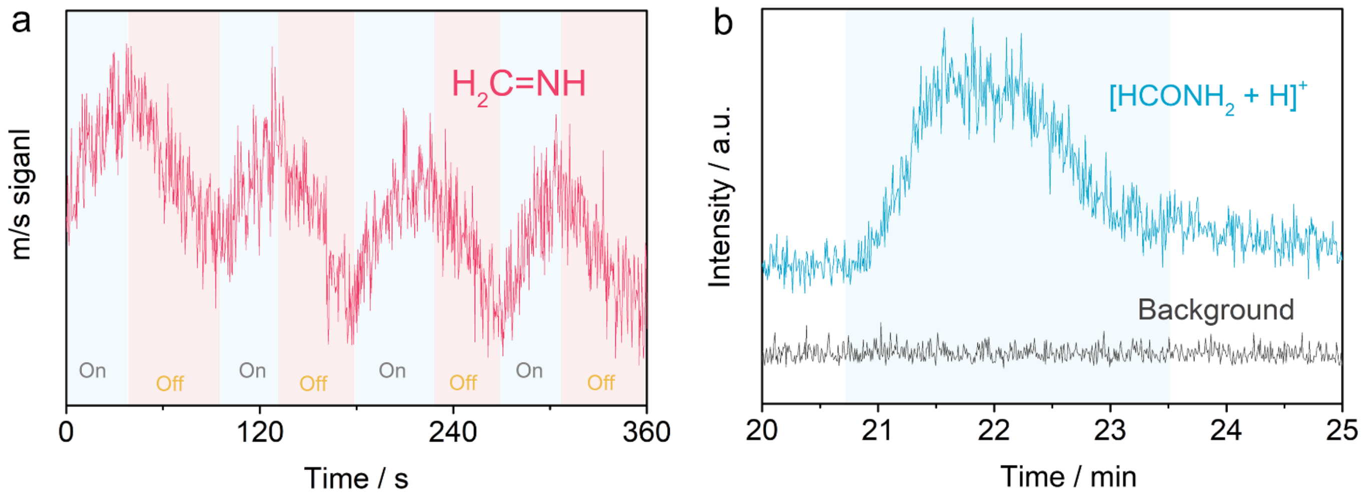

Supplementary Figure S25 │DEMS and LC-MS data. (a) Online DEMS spectrum of $H_2C{=}NH$ intermediate (different colour zones represent a on or off state.); (b) The LC-MS spectrum of formamide product (light blue zone represents the signal.).

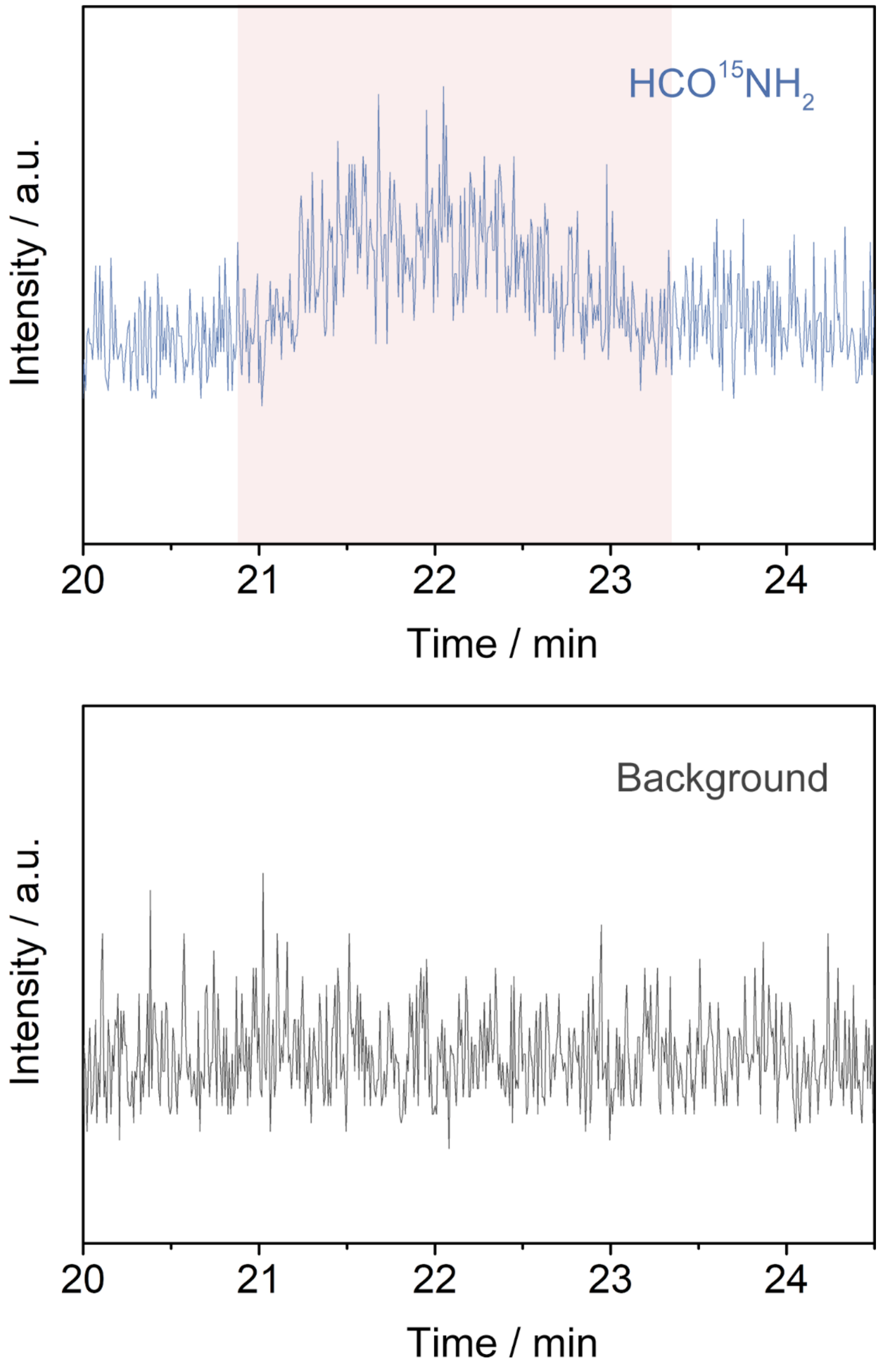

Supplementary Figure 26 │LC-MS data. LC-MS spectrum of $HCO^{15}NH_2$ (light red zone represents the signal)

$HCO^{15}NH_2$ is collected from the isotope-labelling in situ ATR-FTIR measurements and then detected by LC-MS. As shown in Supplementary Fig. 26, $HCO^{15}NH_2$ with 47 molecular weight (molecular weight should plus 1 in the positive ion mode) is well identified.

For adsorbates containing a C-O bond, two kinds of adsorption geometries are considered, i.e, the C-O bond in the adsorbates vertical (Geo-1) and parallel (Geo-2) to the surface, respectively. The numbers of the H bonds with the surface are adjusted for $*CH_3O$. For adsorption of NH_3, two geometries including H (Geo-H) and N (Geo-N1) towards the top sites of metal atoms on the surface, respectively, are considered. For adsorbates of $*NH_2$ and *NH, the metal top and hollow (Geo-N2) sites are token as the binding sites. For $*CH_xNH_y$ (x,y=1,2) adsorbates, the adsorption geometries are designed as C, H or N posing to the surface metal (M) and/or oxygen sites. For *CHN, the geometries are C-N bond parallel (Geo-1) and vertical (Geo-1) to the surface.

Table S1. The binding energies of various adsorbates on α-PtO_2 surface with O stripe characterization.

	Binding energy / eV					
adsorbates	$*CH_3OH$	$*CH_2OH$	$*CH_3O$	*CHOH	$*CH_2O$	*CHO
Geo-1	-0.03	0.10	Decompose	Decompose	1.16	3.24
Geo-2-0	-0.04	0.19	1.44	Decompose	1.13	1.00
Geo-2-1			1.24			
adsorbates	$*NH_3$	$*NH_2$	*NH			
Geo-H	-0.53					
Geo-N1	-1.05	0.70	2.81			
Geo-N2		0.64	2.07			
adsorbates	$*CH_2OHNH_3$	$*CH_2ONH_3$	$*CH_2OHNH_2$	$*CH_2ONH_2$	$*CHOHNH_2$	$*CHONH_2$
Geo-2-0	splitting	splitting	-0.77	Decompose	-1.96	-1.76
Geo-2-1	splitting	splitting	-0.78	-1.76	-1.90	-1.52
adsorbates	$*CH_2NH$	$*CHNH_2$	*CHNH	*CHN		
Geo-C-O/Geo-1	0.42	0.39	0.28	1.01		
Geo-C-M /Geo-2	0.38	0	0.28	1.12		
Geo-H-O			Decompose			

* ‘Decompose’ and ‘splitting’ describe the states of as-indicated adsorbates decomposed with the formation of *H, and $*CH_xO$-NH_3 split to $*CH_xO$ + $*NH_3$.

For $*CH_xOH_y$ (x=1, 2, 3; y=0,1), two kinds of adsorption geometries are considered, i.e, the C-O bond in the adsorbates vertical (Geo-1) and parallel (Geo-2) to the surface, respectively. The numbers of the H bonds with the surface are adjusted for $*CH_3O$ and *CHOH. For adsorption of NH_3, two geometries including H (Geo-H) and N (Geo-N1) towards the top sites of metal atoms on the surface, respectively, are considered. For adsorbates of $*NH_2$ and *NH, the metal top and hollow (Geo-N2) sites are token as the binding sites. For $*CH_xONH_y$ (x,y=1,2), the adsorption geometries are C-N bond vertical (Geo-3) and parallel (Geo-4) to the surface. In Geo-3 cases, three are O (Geo-O-surf) and H (Geo-H-surf) from $*CH_xONH_y$ binding to the surface. For $*CH_xNH_y$ (x,y=1,2) adsorbates, the adsorption geometries are designed as C, H or N posing to the surface metal (M) and/or oxygen sites. For *CHN, the geometries are C-N bond parallel (Geo-1) and vertical (Geo-2) to the surface.

Table S2. The binding energies of various adsorbates on the β-NiOOH(001) surface.

	Binding energy / eV					
adsorbates	$*CH_3OH$	$*CH_2OH$	$*CH_3O$	*CHOH	$*CH_2O$	*CHO
Geo-1	0.81	0.63	0.90	2.10	0.03	Decompose
Ge0-2-0	-0.17	HCOOH	Decompose	2.11	0.02	-1.83
Geo-2-1			1.24	2.81		
adsorbates	$*NH_3$	$*NH_2$	*NH			
Geo-H	-0.94					
Geo-N1	-1.90	0.72	0.57			
Geo-N2		1.25				
adsorbates	$*CH_2OHNH_3$	$*CH_2ONH_3$	$*CH_2OHNH_2$	$*CH_2ONH_2$	$*CHOHNH_2$	$*CHONH_2$
Geo-4	splitting	splitting			Decompose	-1.23
Geo-O-surf				1.08		
Geo-H-surf	splitting	splitting	-1.36	Decompose	Decompose	-1.23
adsorbates	$*CH_2NH$	$*CHNH_2$	*CHNH	*CHN		
Geo-1	2.12	2.15	decompose	0.23		
Geo-2	0	1.37	2.33	-0.64		

* 'Decompose' and 'splitting' describe the states of as-indicated adsorbates decomposed with formation of *H, and $*CH_xO$-NH_3 split to $*CH_xO$ + $*NH_3$.

For $*CH_xOH_y$ (x=1, 2, 3; y=0,1), two kinds of adsorption geometries are considered, i.e, the C-O bond in the adsorbates vertical (Geo-1) and parallel (Geo-2) to the surface, respectively. The numbers of the H bonds with the surface are adjusted for $*CH_3O$ and *CHOH. For adsorption of NH_3, two geometries including H (Geo-H) and N (Geo-N1) towards the top sites of metal atoms on the surface, respectively, are considered. For adsorbates of $*NH_2$ and *NH, the metal top and hollow (Geo-N2) sites are token as the binding sites. For $*CH_xONH_y$ (x,y=1,2), the adsorption geometries are C-N bond vertical (Geo-3) and parallel (Geo-4) to the surface. In Geo-3 cases, three are O (Geo-O-surf) and H (Geo-H-surf) from $*CH_xONH_y$ binding to the surface. For $*CH_xNH_y$ (x,y=1,2) adsorbates, the adsorption geometries are designed as C, H or N posing to the surface metal (M) and/or oxygen sites. For *CHN, the geometries are C-N bond parallel (Geo-1) and vertical (Geo-2) to the surface.

Table S3. The binding energies of various adsorbates on the α-FeOOH(001) surface.

	Binding energy / eV					
adsorbates	$*CH_3OH$	$*CH_2OH$	$*CH_3O$	*CHOH	$*CH_2O$	*COH/*HCO
Geo-1	-0.32	1.22	1.77	3.07	4.16	*HCO Decompose
Ge0-2-0	-0.04	0.64	2.34	3.32	4.40	2.72
Geo-2-1		0.64	1.24	4.54	3.01	*COH Decompose
adsorbates	$*NH_3$	$*NH_2$	*NH			
Geo-H	-0.59					
Geo-N1	-1.29	1.14	3.74			
Geo-N2	-1.30	2.67	6.42			
adsorbates	$*CH_2OH$-$*NH_3$	$*CH_2O$-$*NH_3$	$*CH_2OHNH_2$	$*CH2ONH_2$	$*CHOHNH_2$	$*CHONH_2$
Geo-4	splitting	splitting	0.04		Decompose	4.11
Geo-O-surf				2.04		
Geo-H-surf			-0.39	2.21	Decompose	
adsorbates	$*CH_2NH$	$*CHNH_2$	*CHNH	*CHN		
Geo-1	To Geo-2		Decompose	4.80		
Geo-2	0	0.42	2222	2.71		

* 'Decompose' and 'splitting' describe the states of as-indicated adsorbates decomposed with formation of *H, and $*CH_xO$-NH_3 split to $*CH_xO$ + $*NH_3$.

Table S4. The charged states of the C atom and N atom in the relevant adsorbates on α-PtO_2, β-NiOOH, and α-FeOOH surfaces.

	C in $*CH_2O$	N in NH_3
α-PtO_2	+1.60	-3.00
β-NiOOH	+1.36	-3.00
α-FeOOH	+0.20	-3.00

As shown in Table S4, C in $*CH_2O$ is positively charged in the order of +1.60 e on α-PtO_2 > +1.36 e on β-NiOOH > +0.20 e on α-FeOOH. We, therefore, propose that the barrier energies for the C-N bond-making process by NH_3 nucleophilic attack of $*CH_2O$ are very likely to be in the order of α-PtO_2 > β-NiOOH > α-FeOOH.

Table S5. The summarized properties of α-PtO_2, β-NiOOH, and α-FeOOH as the catalyst for the coupling of methanol and ammonia.

	Applied potential for CH_3OH-$*CH_2O$ step / V	Adsorption energy of $*CH_2ONH_2$ / eV	Reaction energy of $*CH_2O$-$*CHO$ at 0 V /eV	Reaction energy of $*CH_2OHNH_2$ dehydration /eV	Potential dependent step & Reaction energy / eV
α-PtO_2	0.80	-0.23	-0.13	-0.19	$*CH_2OH \rightarrow *CH_2O$ & 1.00
β-NiOOH	1.00	0.91	-2.03	0.55	$*H_2NCH_2OH \rightarrow *H_2NCH_2O$ & 2.51
α-FeOOH	2.25	1.80	-0.73	1.08	$*H_2NCH_2O \rightarrow *H_2NCHO$ & 2.57

Table S6. Control experiments for exploring the reaction pathway.

Entry	C Source	N Source	Current density / mA cm^{-2}	Electrolyte	Formamide / (Faradaic efficiency / Partial current density)
1	CH_3OH	NH_3	100	$NaHCO_3$	32.70 % / 32.70 mA cm^{-2}
2	HCOH	NH_3	100	$NaHCO_3$	4.11 % / 4.11 mA cm^{-2}
3	$HCOO^-$	NH_3	100	$NaHCO_3$	Null

Supplementary Note 1

To evaluate the economic market potential of sustainable formamide electrosynthesis using methanol and ammonia as the feedstocks, we perform a techno-economic analysis (TEA) using a modified model proposed by Sargent *et al.*[12,13]. Supplementary Fig. 27 lists the components to calculate the total basic manufacture costs and product dividends. In this approach, the prices consist of two parts: the costs for manufacture and the special dividends. The costs for manufacture are assumed to constitute catalyst costs, separation device costs, electricity costs, separation costs, feedstock costs, and operation costs. The special dividends are constituted of hydrogen dividend, sodium formate dividend, sodium nitrate dividend, and sodium nitrite dividend.

Here, we carry out a TEA calculation for the case where the optimized condition of the flow cell is performed. Below is a full list of the assumptions made:

1. Catalyst costs are composed of two parts: Pt and Ti. We assume a 1 m^2 area of the catalyst is used for electrolysis with a lifetime of 10 years. Separation device costs are assumed to be 10 % of the catalyst cost. The market prices of Pt and Ti are 52 $ g^{-1} and 0.01 $ g^{-1}, respectively.

2. Electricity cost is assumed as 0.03 $ kWh^{-1} based on recently reported work.

3. Separation costs include the free feedstocks (methanol and ammonia) separation and products (formamide, sodium formate, hydrogen, nitrate, and nitrite) separation. The total separation costs are assumed to be 30 % of the electricity cost.

4. Feedstock costs include the methanol and ammonia reactants. The prices of methanol and 20 % ammonia are 400 $ t^{-1} and 150 $ t^{-1}, respectively.

5. Operation costs are assumed as 10 % of catalyst costs and separation device cost.

6. The operational time of electrolysis is assumed to be 80 % of a day (19.2 h).

7. Electrolysis will convert 200 tonnes of methanol to 281.25 tonnes of formamide per day. The selectivity rates of the starting methanol and ammonia to formamide are 76.8 % and 60.8 %, respectively. Thus, this will consume 260.4 tonnes of methanol and 873.8 tonnes of 20 % ammonia, respectively.

8. The prices of formamide, sodium formate, hydrogen, sodium nitrate and sodium nitrite are assumed as 3000 $ t^{-1}, 500 $ t^{-1}, 1900 $ t^{-1}, 462 $ t^{-1} and 600 $ t^{-1}, respectively.

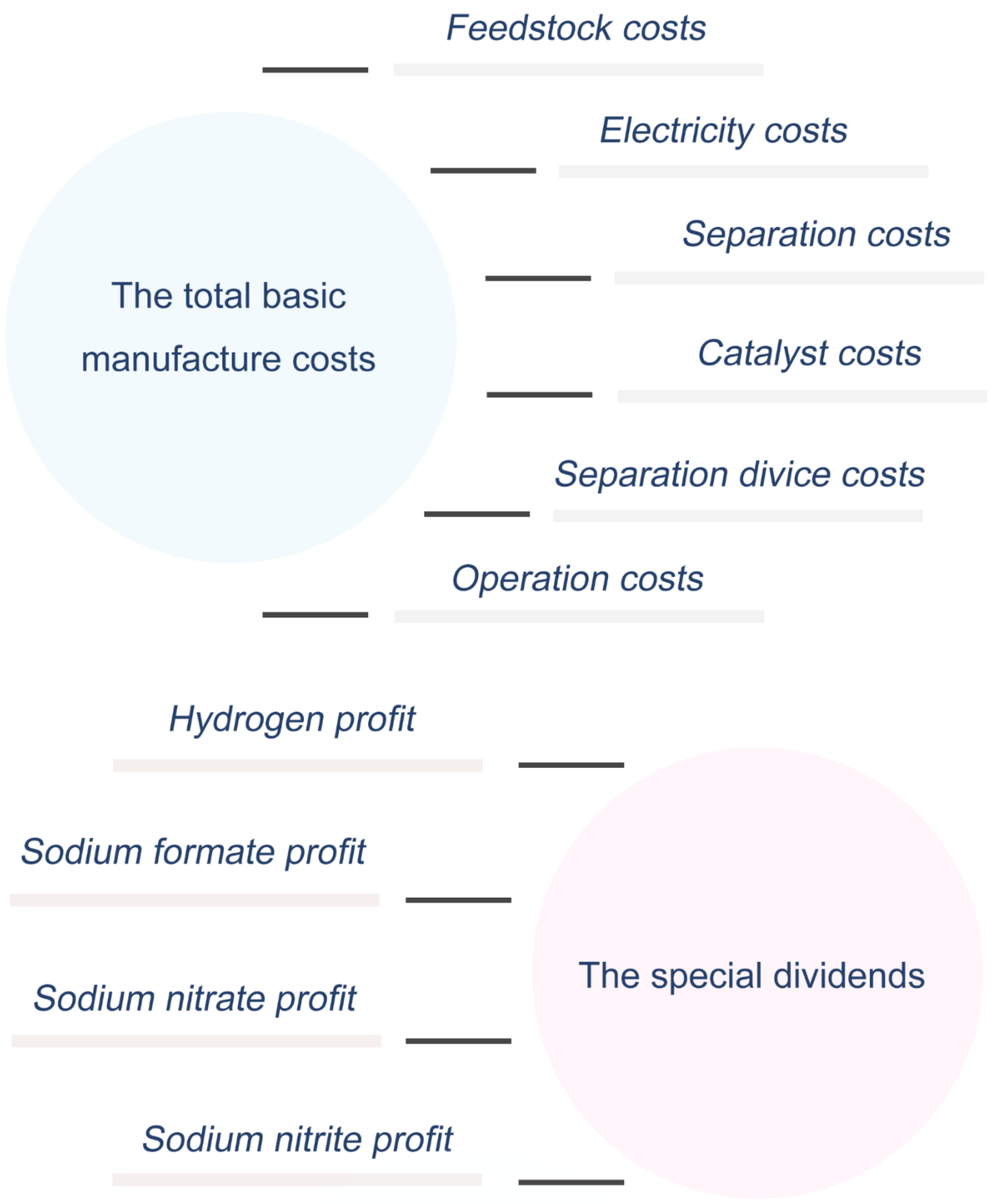

Supplementary Figure S27 │Techno-economic analysis. Model used for calculating the techno-economic analysis of sustainable formamide electrosynthesis using methanol and ammonia as the feedstocks.

Calculations details

The total basic manufacture costs

(1) Feedstock costs

Feedstock costs per tonne = (Cost of methanol × Mass of methanol reacted + Cost of ammonia × Mass of ammonia reacted) / Mass of formamide produced per day

$$[(400 \times 260.4 + 150 \times 873.8) / 281.25]\ \$ = 836.4\ \$$$

(2) Electricity costs

We initially calculate the total charge needed to oxidize 200 tonnes of methanol per day and assume the energy losses is 20 %.

$$Q = (\text{Mass of methanol consumed} / \text{Molar mass of methanol} \times N \times F) / \text{Faradaic efficiency} / (1 - 20\ \%)$$

Where Q is the total charge, F is the Faraday constant (96485 C), N is 4 since methanol-to-formamide is a four-electron reaction process.

$$[(200 \times 10^6) / 32 \times 4 \times 96485 / 0.3788 / 0.8]\ \text{C} = 7.96 \times 10^{12}\ \text{C}$$

The corresponding current:

$$I = Q / t$$

$$[7.96 \times 10^{12} / (19.2 \times 3600)]\ \text{A} = 115158532.8\ \text{A}$$

The corresponding power:

$$P = UI$$

$$(1.3 \times 115158532.8)\ \text{W} = 149706092.7\ \text{W}$$

The energy consumption per day:

$$\text{Energy consumption per day} = P \times t$$

$$(149706092.7 / 1000 \times 19.2)\ \text{KW h} = 2874356.979\ \text{KW h}$$

Thus, the electricity cost per tonne for formamide generation:

Electricity costs per tonne = (Energy consumption per day × Cost per kWh) / Mass of formamide per day

$$[(2874356.979 \times 0.03) / 281.25]\ \$ = 306.6\ \$$$

(3) The total separation costs

The total separation costs per tonne = Electricity cost per tonne × 30 %

$$(306.6 \times 30\ \%)\ \$ = 91.98\ \$$$

(4) Catalyst costs and Separation device costs

Catalyst costs per square meter

The thickness of Pt on Ti substrate is 480 nm (Figure S5c). The density of Pt is 21.45 g cm^{-3}.

$$\text{Pt price } (m^2) = \text{The volume of Pt} / \text{The density of Pt} \times \text{The price of Pt}$$

$$\{[(480 \times 2) \times 10^{-9} \times 1] / (21.45 \times 10^{6}) \times 52\} \$ = 1070.784 \$$$

The thickness of Ti substrate is 0.2 mm. The density of Ti is 4.5 g cm^{-2}.

$$\textit{Ti price } (m^2) = \textit{The volume of Ti / The density of Ti} \times \textit{The price of Ti}$$

$$\{(0.2 \times 10^{-3} \times 1) / (4.5 \times 10^{6}) \times 0.01\} \$ = 9.012 \$$$

$$\textit{Catalyst costs } (m^2) = \textit{Pt price } (m^2) + \textit{Ti price } (m^2)$$

$$(1070.784 + 9.012) \$ = 1079.796 \$.$$

We evaluate the catalyst area needed. Based on the applied potential (1.3 V) and operating current density (33.36 mA cm^{-2}), we can calculate the area of catalyst costs needed.

The area of catalyst costs needed = the current per day / the operating current density

$$[115158532.8 / (33.36 \times 10)] \text{ m}^2 = 345199.4389 \text{ m}^2$$

Catalyst costs = The area of catalyst costs needed × Catalyst costs per square meter

$$(345199.4388 \times 1079.796) \$ = 372744973.3 \$$$

Separation device costs = Catalyst costs × 10 %

$$(372744973.3 \times 10 \%) \$ = 37274497.33 \$$$

The costs for Catalyst and Separation device per tonne = (Catalyst costs + Separation device cost) / (lifetime of electrolyzer / Mass of formamide produced per day

$$[(372744973.3 + 37274497.33) / (10 \times 365) / 281.25] \$ = 399.41 \$$$

(5) <u>Operation costs</u>

Operation costs per tonne = The costs for Catalyst and Separation devices per tonne × 10 %

$$(399.41 \times 10 \%) \$ = 39.941 \$$$

<u>The costs for manufacture</u>

The costs for manufacture per tonne = Feedstock costs per tonne + Electricity cost per tonne + The total separation costs per tonne + The costs for Catalyst and Separation device per tonne + Operation cost per tonne

$$(836.4 + 306.6 + 91.98 + 399.41 + 39.941) \$ = 1674.331 \$$$

<u>The profit</u>

The profit per tonne from this electrosynthesis can be calculated based on the market price of formamide.

The profit = The market price of formamide - The costs for manufacture

$$(3000 - 1674.331) \$ = 1325.669 \$$$

The special dividends

(1) <u>Hydrogen profit</u>

Hydrogen is generated at the cathode and assumed as 100 % Faradaic efficiency.

$$\textit{Mass of hydrogen produced per day} = Q \times FE_{Hydrogen} / F / N * \textit{Molar mass of hydrogen}$$

$$[(7.96 \times 10^{12} \times 100\ \% / 96485 / 2 \times 2) \times 10^{-6}]\ t = 82.5\ t$$

$$\textit{Profit of hydrogen per day} = \textit{Mass of hydrogen produced per day} \times \textit{Market price}$$

$$(82.5 \times 1900)\ \$ = 156750\ \$$$

$$\textit{Profit of hydrogen per tonne of formamide} = \textit{Profit of hydrogen per day} / \textit{Mass of formamide produced per day}$$

$$(156750 / 281.25)\ \$ = 557.33\ \$$$

(2) Sodium formate profit

Sodium formate is generated at the anode and assumed as a 3.47 % Faradaic efficiency.

$$\textit{Mass of sodium formate produced per day} = Q \times FE_{Sodium\ formate} / F / N * \textit{Molar mass of sodium formate}$$

$$[(7.96 \times 10^{12} \times 3.47\ \% / 96485 / 4 \times 68) \times 10^{-6}]\ t = 48.67\ t$$

$$\textit{Profit of sodium formate per day} = \textit{Mass of sodium formate produced per day} \times \textit{Market price}$$

$$(48.67 \times 500)\ \$ = 24335\ \$$$

$$\textit{Profit of sodium formate per tonne of formamide} = \textit{Profit of sodium formate per day} / \textit{Mass of formamide produced per day}$$

$$(24335 / 281.25)\ \$ = 86.52\ \$$$

(3) Sodium nitrate profit

Sodium nitrate is generated at the anode and assumed as a 8.71 % Faradaic efficiency.

$$\textit{Mass of sodium nitrate produced per day} = Q \times FE_{Sodium\ nitrate} / F / N * \textit{Molar mass of sodium nitrate}$$

$$[(7.96 \times 10^{12} \times 8.71\ \% / 96485 / 8 \times 85) \times 10^{-6}]\ t = 76.35\ t$$

$$\textit{Profit of sodium nitrate per day} = \textit{Mass of sodium nitrate produced per day} \times \textit{Market price}$$

$$(76.35 \times 462)\ \$ = 35273.7\ \$$$

$$\textit{Profit of sodium nitrate per tonne of formamide} = \textit{Profit of sodium nitrate per day} / \textit{Mass of formamide produced per day}$$

$$(35273.7 / 281.25)\ \$ = 125.42\ \$$$

(4) Sodium nitrite profit

Sodium nitrite is generated at the anode and assumed as a 3.16 % Faradaic efficiency.

$$\textit{Mass of sodium nitrite produced per day} = Q \times FE_{Sodium\ nitrate} / F / N * \textit{Molar mass of sodium nitrate}$$

$$[(7.96 \times 10^{12} \times 3.16\ \% / 96485 / 6 \times 69) \times 10^{-6}]\ t = 29.98\ t$$

$$\textit{Profit of sodium nitrite per day} = \textit{Mass of sodium nitrite produced per day} \times \textit{Market price}$$

$$(29.98 \times 600)\ \$ = 17988\ \$$$

$$\textit{Profit of sodium nitrite per tonne of formamide} = \textit{Profit of sodium nitrite per day} / \textit{Mass of formamide produced per day}$$

$$(17988 / 281.25)\ \$ = 63.96\ \$$$

The total special dividends

The total special dividends per tonne of formamide = Hydrogen profit per tonne of formamide + Sodium formate profit per tonne of formamide + Sodium nitrate profit per tonne of formamide + Sodium nitrite profit per tonne of formamide

(557.33 + 86.52 + 125.42 + 63.96) $ = 833.23 $

The total profits

The total profits per tonne from this electrosynthesis strategy can be calculated based on the market price of formamide and the total special dividends.

The total profits = The market price of formamide - *The costs for manufacture + The total special dividends*

(3000 - 1674.331 + 833.23) $ = 2158.899 $

Supplementary References

1. Yamaguchi, K., Kobayashi, H., Oishi, T. & Mizuno, N. Heterogeneously catalyzed synthesis of primary amides directly from primary alcohols and aqueous ammonia. *Angew. Chem. Int. Ed.* **51**, 544-547 (2012).

2. Mihaylov, M., Ivanova, E., Aleksandrov, H., Petkov, P., Vayssilov, G. & Hadjiivanov, K. Species formed during NO adsorption and NO + O_2 co-adsorption on ceria: A combined FTIR and DFT study, *Mol. Catal.* **451**, 114-124 (2018).

3. Meng, N., Zhou, W., Yu, Y., Liu, Y. & Zhang, B. Superficial hydroxyl and amino groups synergistically active polymeric carbon nitride for CO_2 electroreduction. *ACS Catal.* **9**, 10983-10989 (2019).

4. Kresse, G. & Furthmüller, J. Efficient iterative schemes for ab initio total-energy calculations using a plane-wave basis set. *Phys. Rev. B* **54**, 11169-11186 (1996).

5. Perdew, J., Kieron Burke, K. & Ernzerhof, M. Generalized gradient approximation made simple. *Phys. Rev. Lett.* **77**, 3865-3868 (1996).

6. Kresse, G. & Joubert, D. From ultrasoft pseudopotentials to the projector augmented-wave method. *Phys. Rev. B* **59**, 1758-1775 (1999).

7. Monkhorst, H. & Pack, J. Special points for Brillouin-zone integrations. *Phys. Rev. B* **13**, 5188-5192 (1976).

8. Methfessel, M & Paxton, A. High-precision sampling for Brillouin-zone integration in metals. *Phys. Rev. B* **40**, 3616-3621 (1989).

9. Cohen, A., Mori-Sánchez, P. & Yang, W. Insights into current limitations of density functional theory. *Science* **321**, 792-794 (2008).

10. Mathew, K., Sundararaman, R., Letchworth-Weaver, K., Arias, T. & Hennig, R. Implicit solvation model for density-functional study of nanocrystal surfaces and reaction pathways. *J. Chem. Phys*. **140**, 084106 (2014).

11. Mathew, K., Kolluru, V., Mula, S., Steinmann, S. & Hennig, R. Implicit self-consistent electrolyte model in plane-wave density-functional theory. *J. Chem. Phys.* **151**, 234101 (2019).

12. Lum, Y. *et al.* Tuning OH binding energy enables selective electrochemical oxidation of ethylene to ethylene glycol. *Nat Catal* **3,** 14-22 (2020).

13. Leow, W. *et al.* Chloride-mediated selective electrosynthesis of ethylene and propylene oxides at high current density. *Science* **368**, 1228-1233 (2020).
